# Supplementary figures and images for: The actin binding protein profilin 1 localizes inside mitochondria and is critical for their function
Source: EMBO Rep. 2024 Jul 18;25(8):8. doi: 10.1038/s44319-024-00209-3 (PMC11316047; doi:10.1038/s44319-024-00209-3)

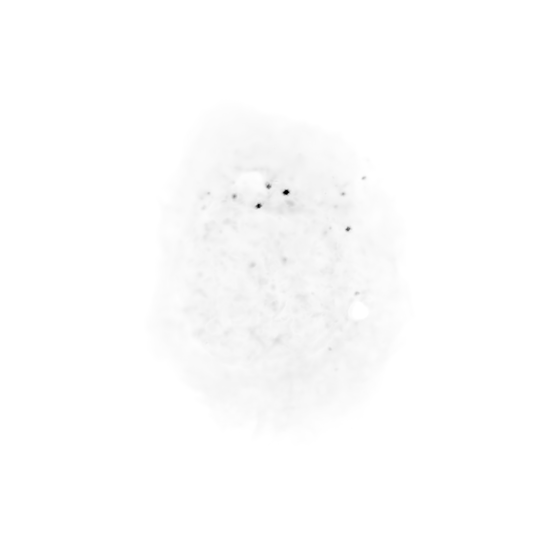

Supplement: Supplementary file 2 — Source data Fig. 1 [file 44319_2024_209_MOESM2_ESM.zip › Figure 1/1B/Control + GFP.tif]

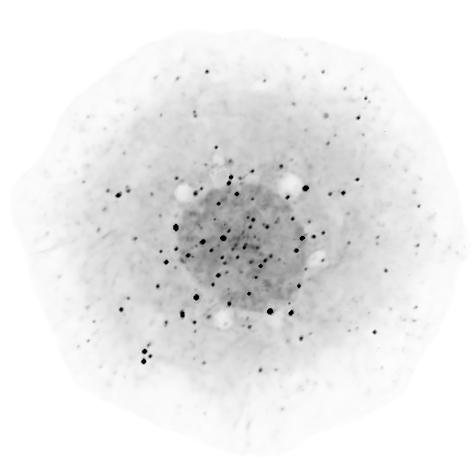

Supplement: Supplementary file 2 — Source data Fig. 1 [file 44319_2024_209_MOESM2_ESM.zip › Figure 1/1B/PFN1 KO + GFP.tif]

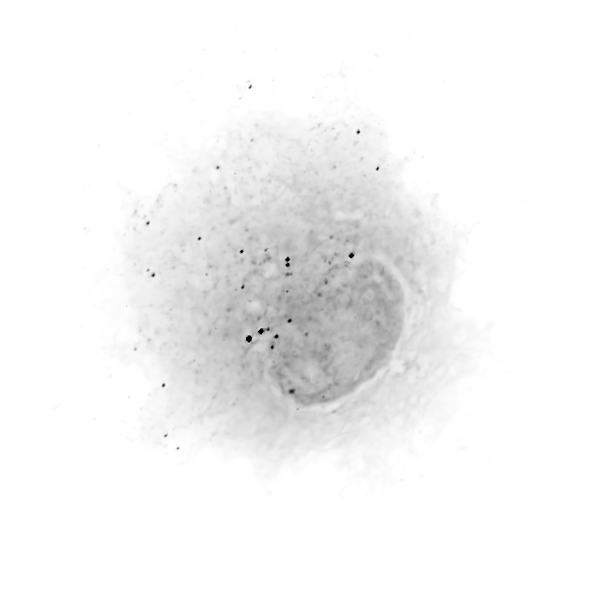

Supplement: Supplementary file 2 — Source data Fig. 1 [file 44319_2024_209_MOESM2_ESM.zip › Figure 1/1B/PFN1 KO + GFP-PFN1.tif]

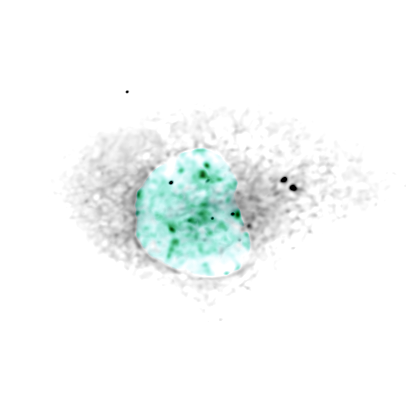

Supplement: Supplementary file 2 — Source data Fig. 1 [file 44319_2024_209_MOESM2_ESM.zip › Figure 1/1D/Control+DMSO.tif]

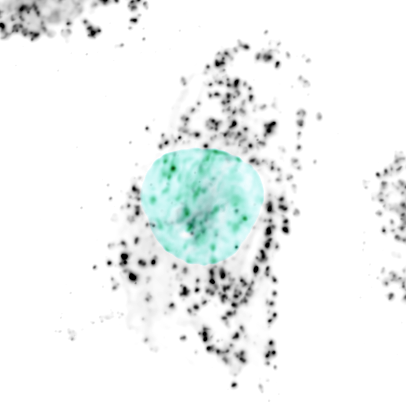

Supplement: Supplementary file 2 — Source data Fig. 1 [file 44319_2024_209_MOESM2_ESM.zip › Figure 1/1D/Control+Baf.tif]

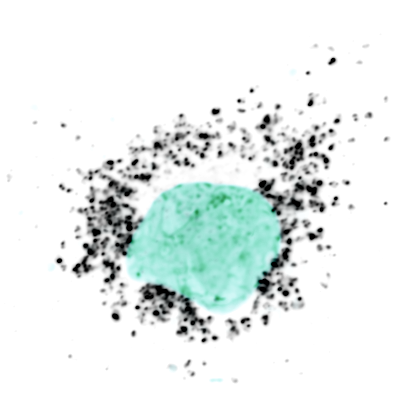

Supplement: Supplementary file 2 — Source data Fig. 1 [file 44319_2024_209_MOESM2_ESM.zip › Figure 1/1D/PFN1 KO+Baf.tif]

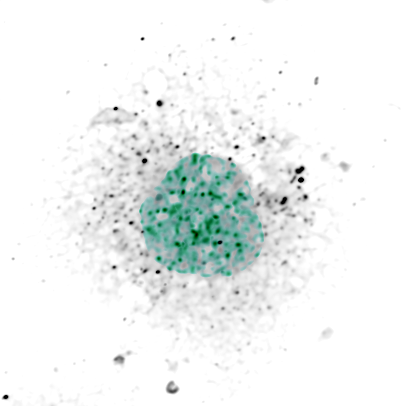

Supplement: Supplementary file 2 — Source data Fig. 1 [file 44319_2024_209_MOESM2_ESM.zip › Figure 1/1D/PFN1 KO+DMSO.tif]

Fig. 1C

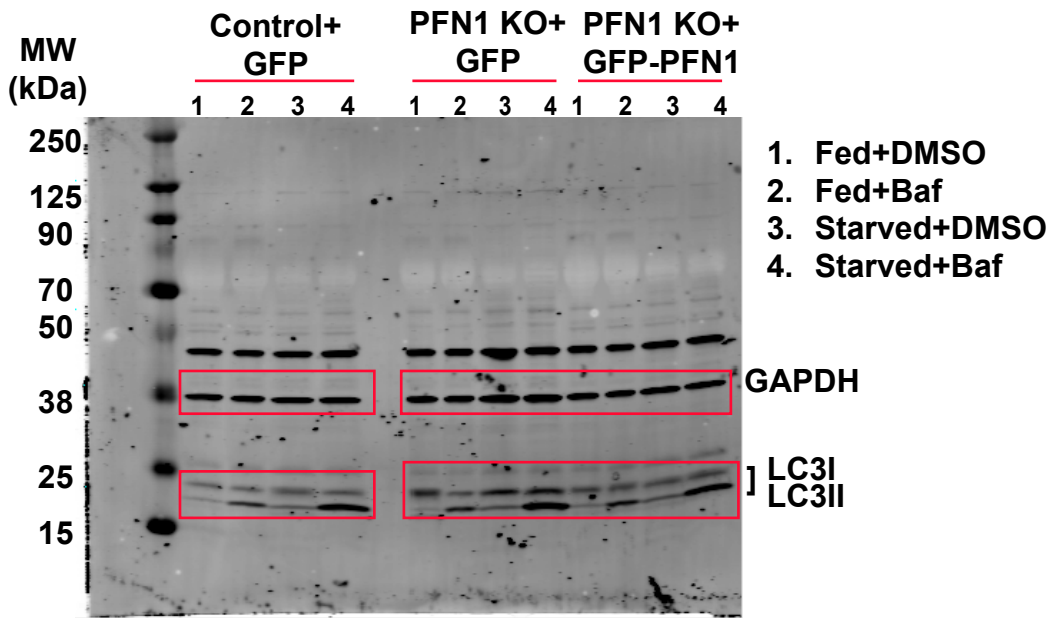

Supplement: Supplementary file 2 — Source data Fig. 1 [file 44319_2024_209_MOESM2_ESM.zip › Figure 1/1C/1C western.pdf]

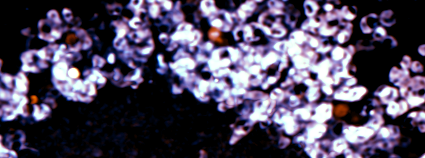

Supplement: Supplementary file 3 — Source data Fig. 2 [file 44319_2024_209_MOESM3_ESM.zip › Figure 2/2F/Control + DMSO COX8-GFP-mCherry inset.tif]

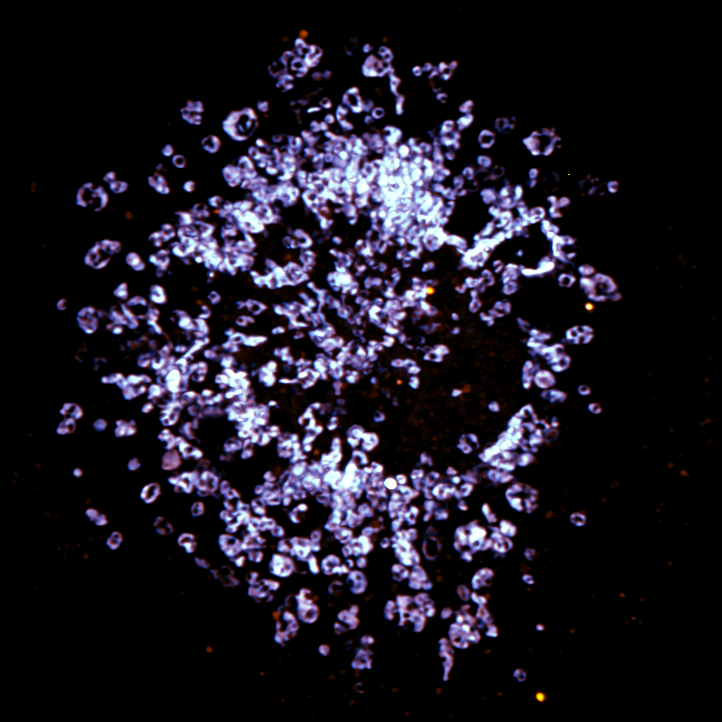

Supplement: Supplementary file 3 — Source data Fig. 2 [file 44319_2024_209_MOESM3_ESM.zip › Figure 2/2F/Control + LatA COX8-GFP-mCherry.tif]

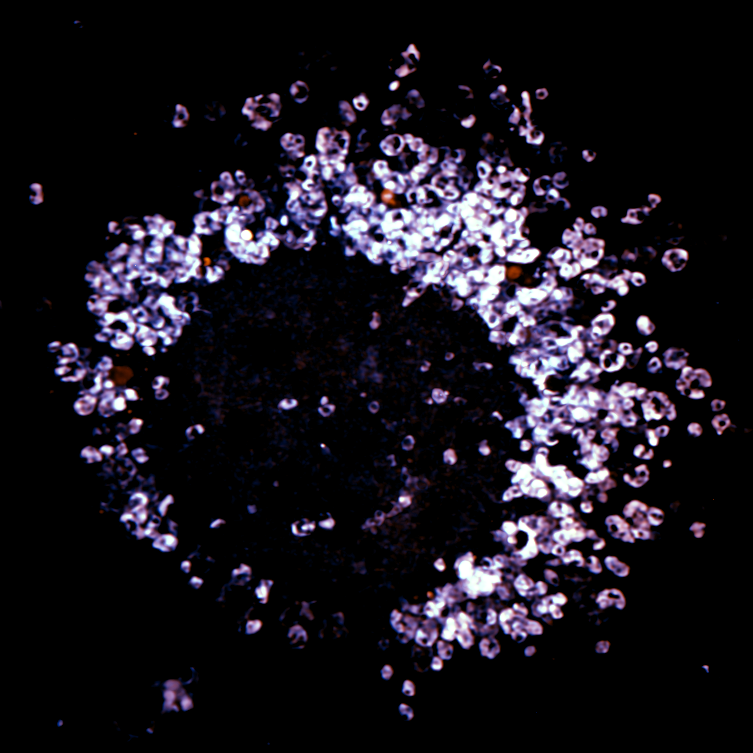

Supplement: Supplementary file 3 — Source data Fig. 2 [file 44319_2024_209_MOESM3_ESM.zip › Figure 2/2F/Control + DMSO COX8-GFP-mCherry.tif]

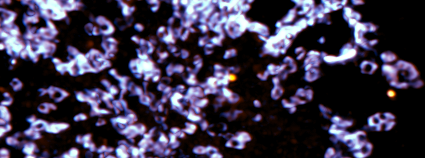

Supplement: Supplementary file 3 — Source data Fig. 2 [file 44319_2024_209_MOESM3_ESM.zip › Figure 2/2F/Control + LatA COX8-GFP-mCherry inset.tif]

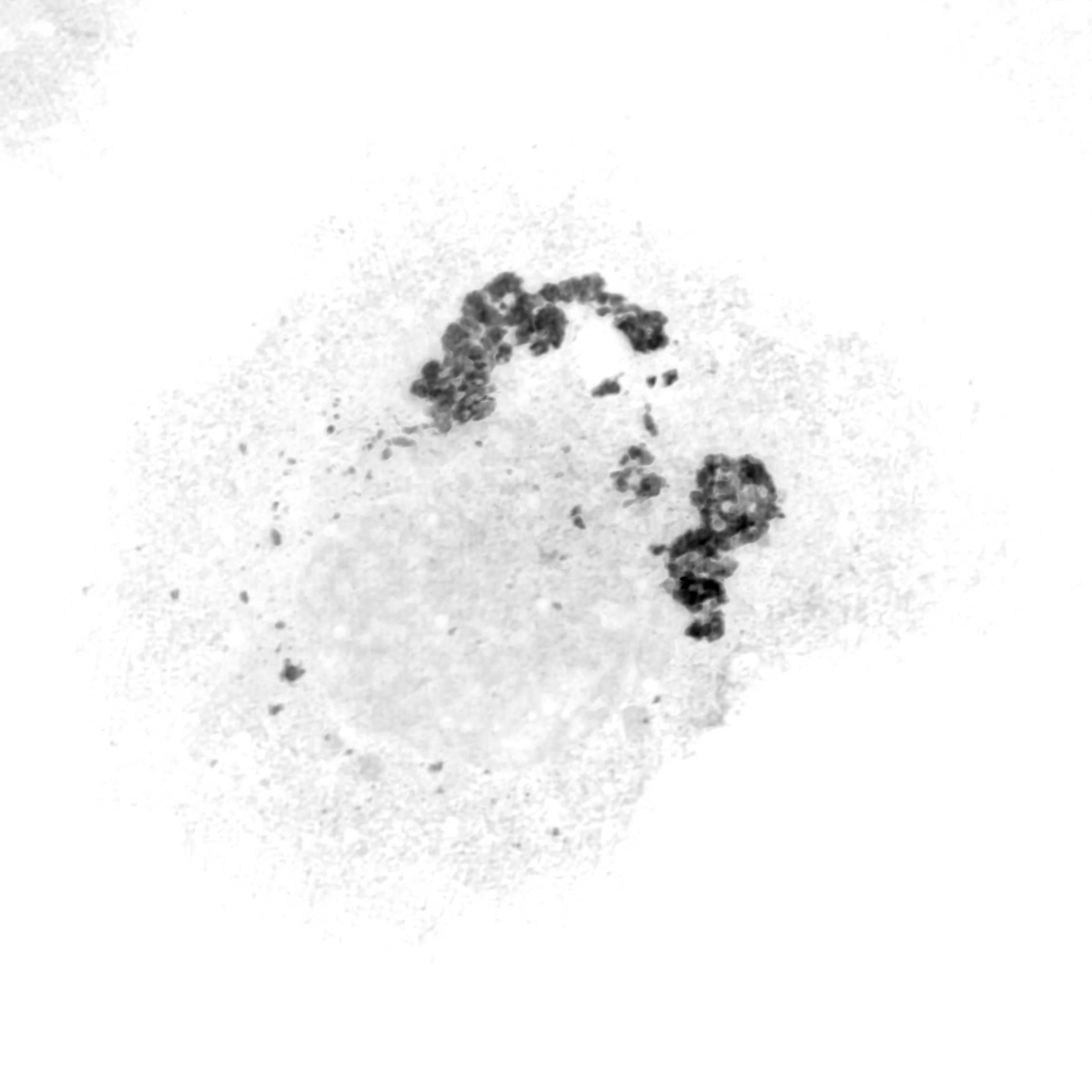

Supplement: Supplementary file 3 — Source data Fig. 2 [file 44319_2024_209_MOESM3_ESM.zip › Figure 2/2C/PFN1 KO mCherry-Parkin.tif]

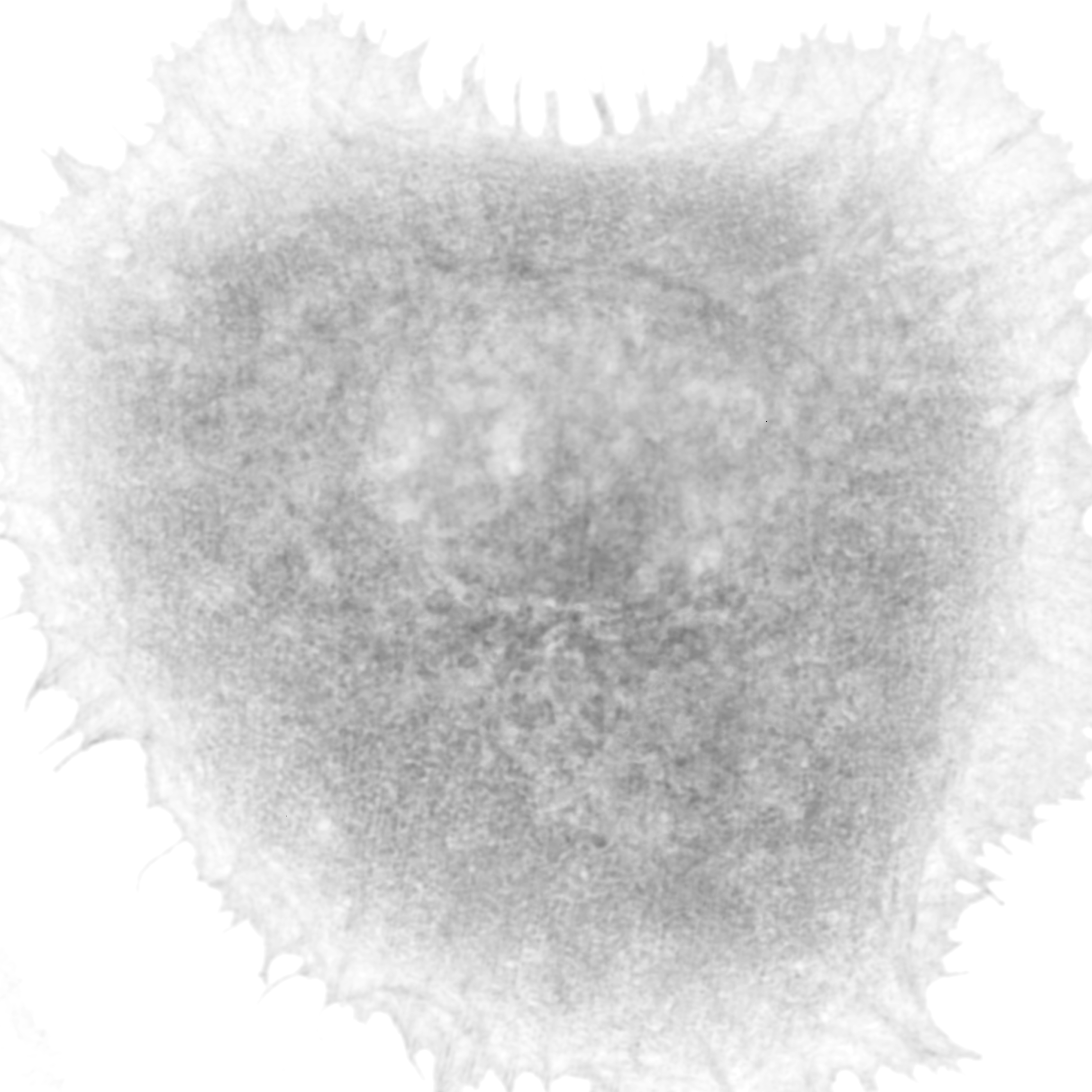

Supplement: Supplementary file 3 — Source data Fig. 2 [file 44319_2024_209_MOESM3_ESM.zip › Figure 2/2C/Control mCherry-Parkin.tif]

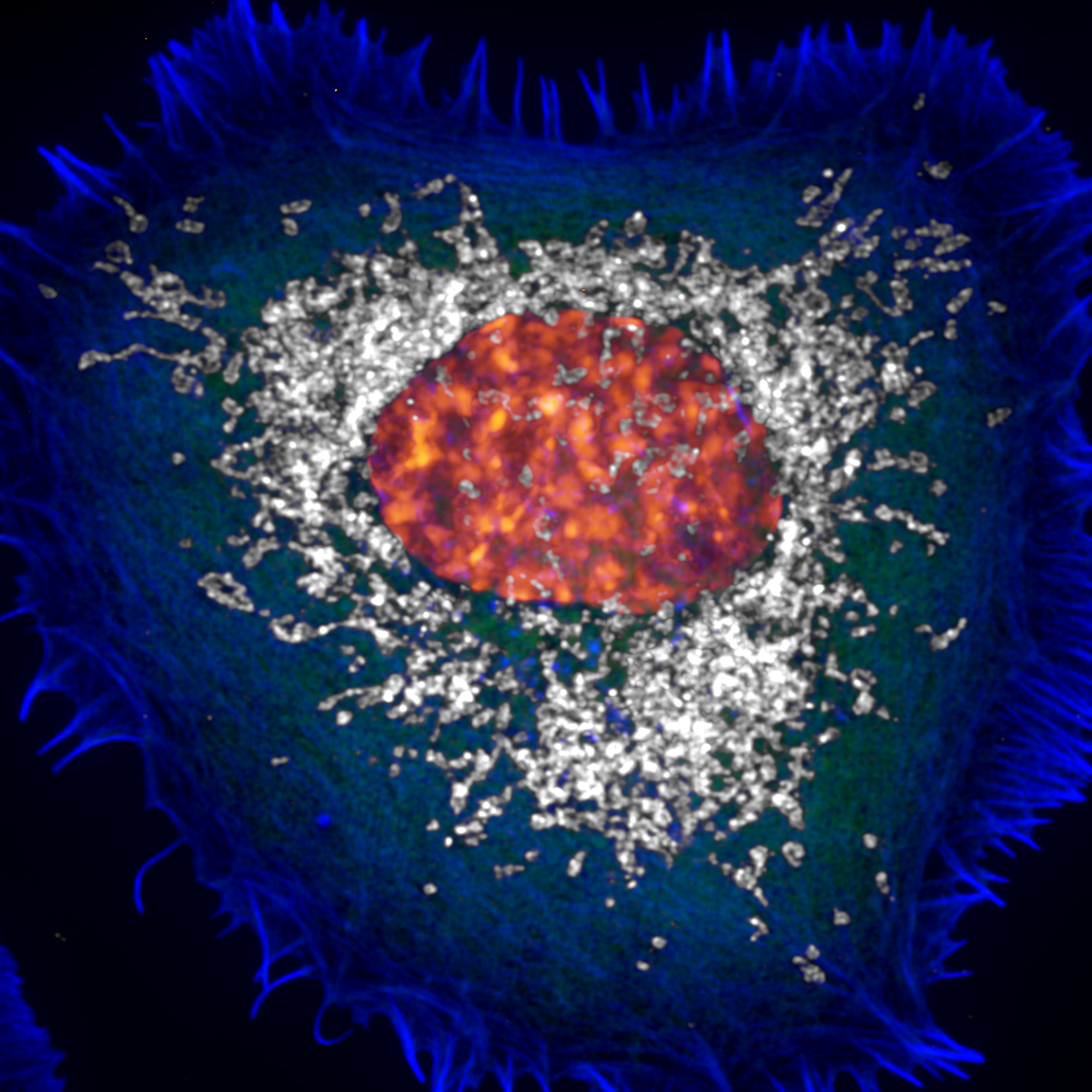

Supplement: Supplementary file 3 — Source data Fig. 2 [file 44319_2024_209_MOESM3_ESM.zip › Figure 2/2C/Control Merge.tif]

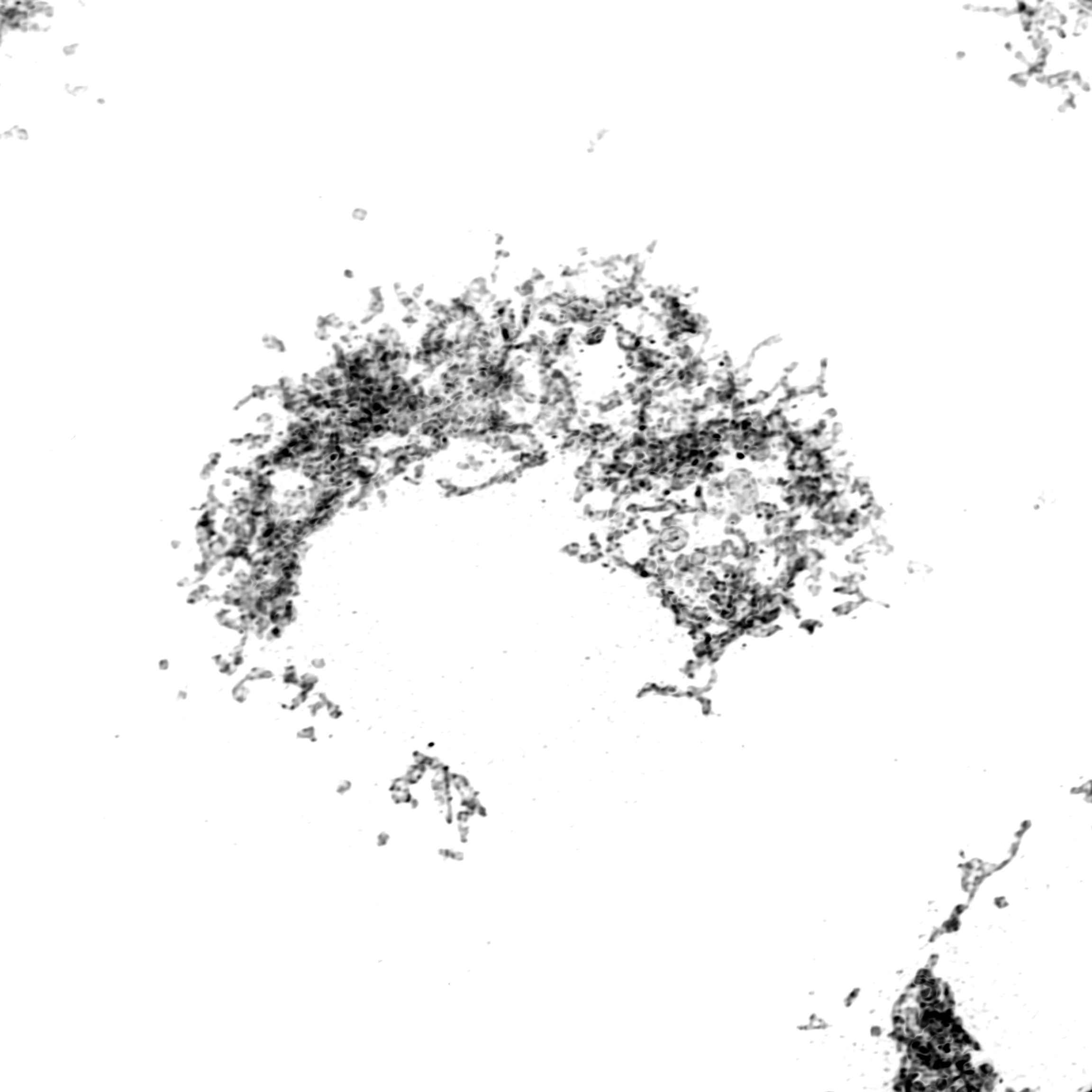

Supplement: Supplementary file 3 — Source data Fig. 2 [file 44319_2024_209_MOESM3_ESM.zip › Figure 2/2C/PFN1 KO TOM20.tif]

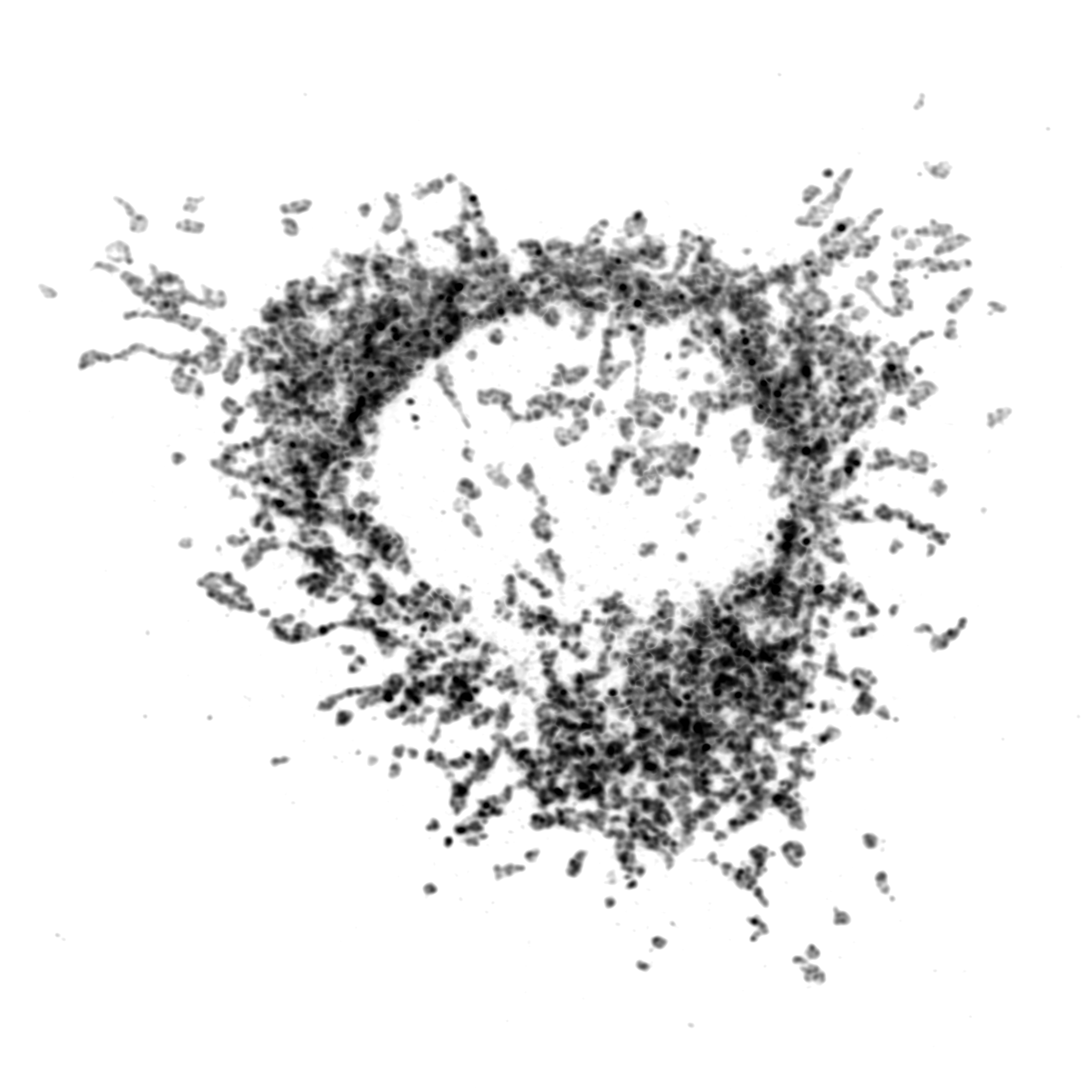

Supplement: Supplementary file 3 — Source data Fig. 2 [file 44319_2024_209_MOESM3_ESM.zip › Figure 2/2C/Control TOM20.tif]

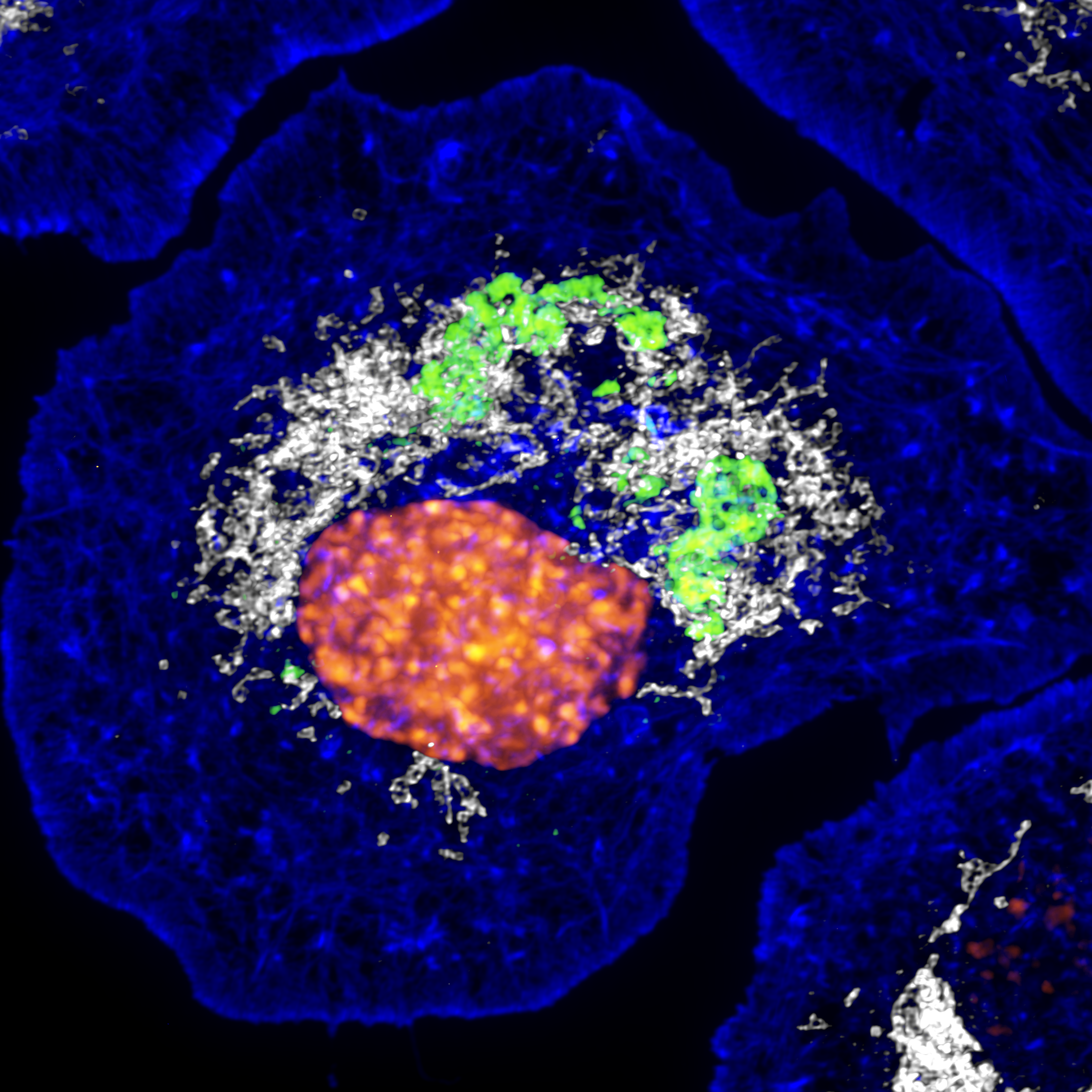

Supplement: Supplementary file 3 — Source data Fig. 2 [file 44319_2024_209_MOESM3_ESM.zip › Figure 2/2C/PFN1 KO Merge.tif]

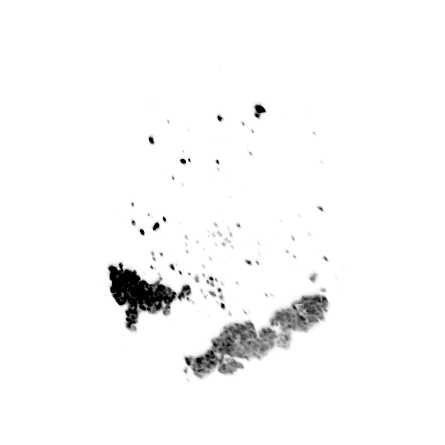

Supplement: Supplementary file 3 — Source data Fig. 2 [file 44319_2024_209_MOESM3_ESM.zip › Figure 2/2D/PFN1 KO +DMSO GFP-p62.tif]

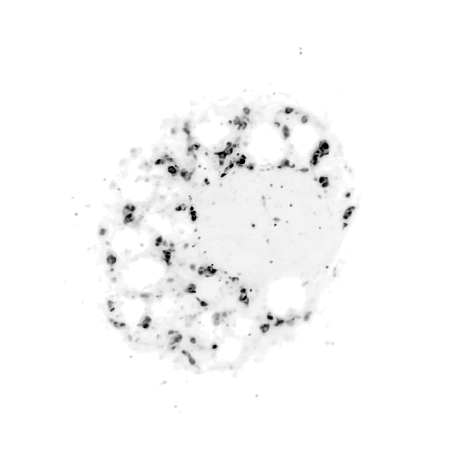

Supplement: Supplementary file 3 — Source data Fig. 2 [file 44319_2024_209_MOESM3_ESM.zip › Figure 2/2D/PFN1 KO + FCCP GFP-p62.tif]

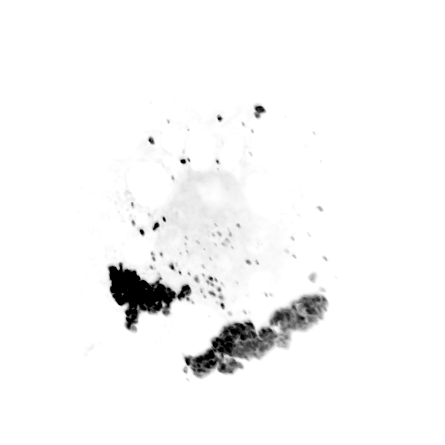

Supplement: Supplementary file 3 — Source data Fig. 2 [file 44319_2024_209_MOESM3_ESM.zip › Figure 2/2D/PFN1 KO +DMSO mCherry-Parkin.tif]

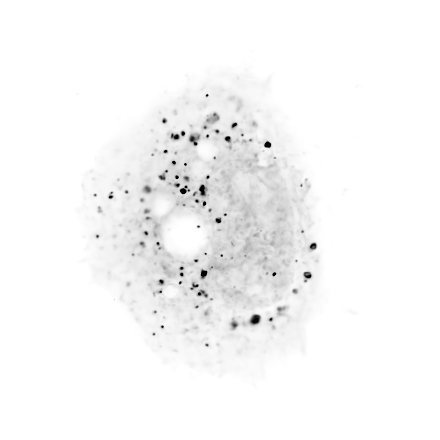

Supplement: Supplementary file 3 — Source data Fig. 2 [file 44319_2024_209_MOESM3_ESM.zip › Figure 2/2D/Control + FCCP GFP-p62.tif]

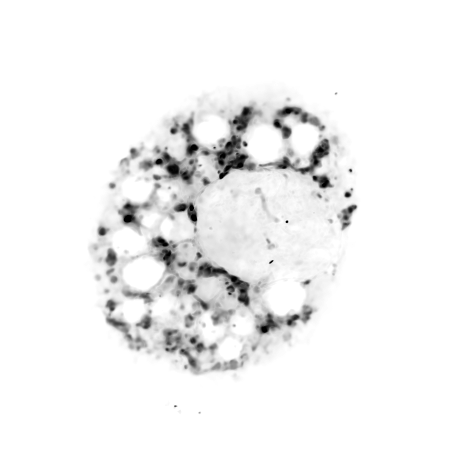

Supplement: Supplementary file 3 — Source data Fig. 2 [file 44319_2024_209_MOESM3_ESM.zip › Figure 2/2D/PFN1 KO + FCCP mCherry-Parkin.tif]

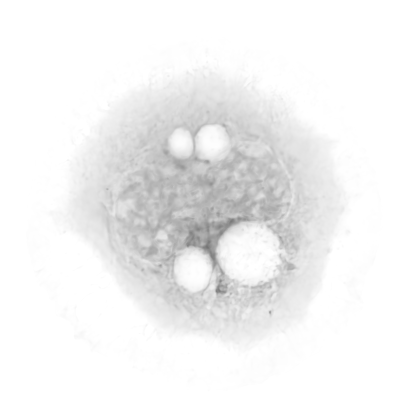

Supplement: Supplementary file 3 — Source data Fig. 2 [file 44319_2024_209_MOESM3_ESM.zip › Figure 2/2D/Control + DMSO mCherry-Parkin.tif]

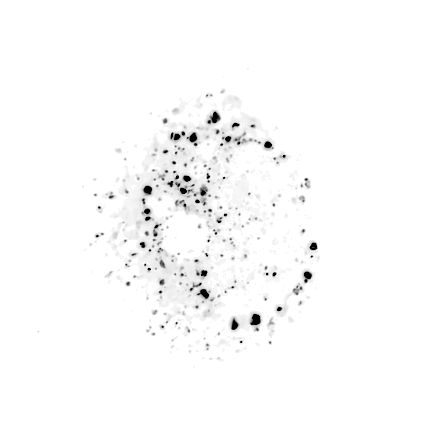

Supplement: Supplementary file 3 — Source data Fig. 2 [file 44319_2024_209_MOESM3_ESM.zip › Figure 2/2D/Control + FCCP mCherry-Parkin.tif]

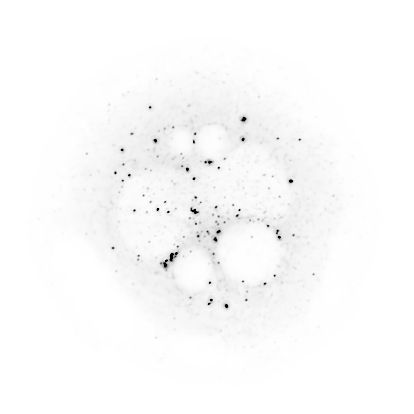

Supplement: Supplementary file 3 — Source data Fig. 2 [file 44319_2024_209_MOESM3_ESM.zip › Figure 2/2D/Control + DMSO GFP-p62.tif]

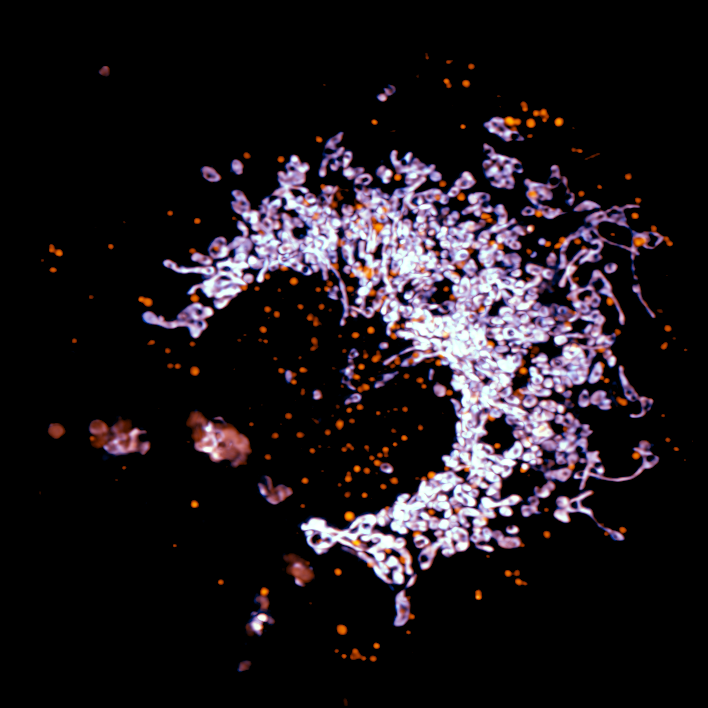

Supplement: Supplementary file 3 — Source data Fig. 2 [file 44319_2024_209_MOESM3_ESM.zip › Figure 2/2E/PFN1 KO COX8-GFP-mCherry.tif]

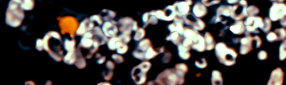

Supplement: Supplementary file 3 — Source data Fig. 2 [file 44319_2024_209_MOESM3_ESM.zip › Figure 2/2E/Control COX8-GFP-mCherry inset.tif]

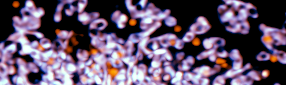

Supplement: Supplementary file 3 — Source data Fig. 2 [file 44319_2024_209_MOESM3_ESM.zip › Figure 2/2E/PFN1 KO COX8-GFP-mCherry inset.tif]

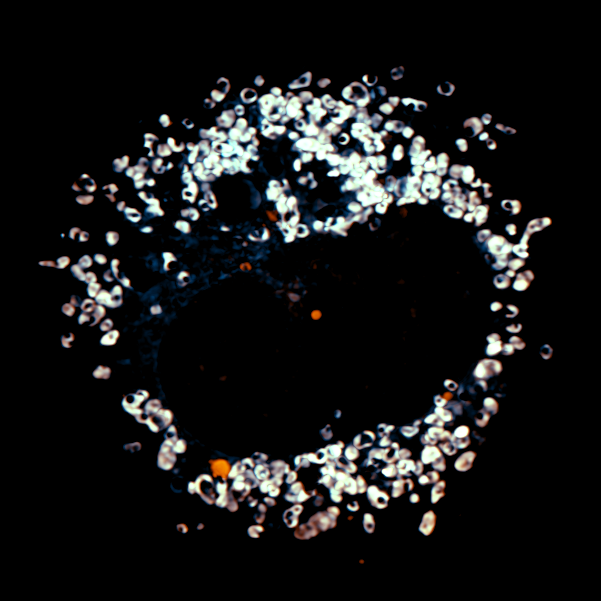

Supplement: Supplementary file 3 — Source data Fig. 2 [file 44319_2024_209_MOESM3_ESM.zip › Figure 2/2E/Control COX8-GFP-mCherry.tif]

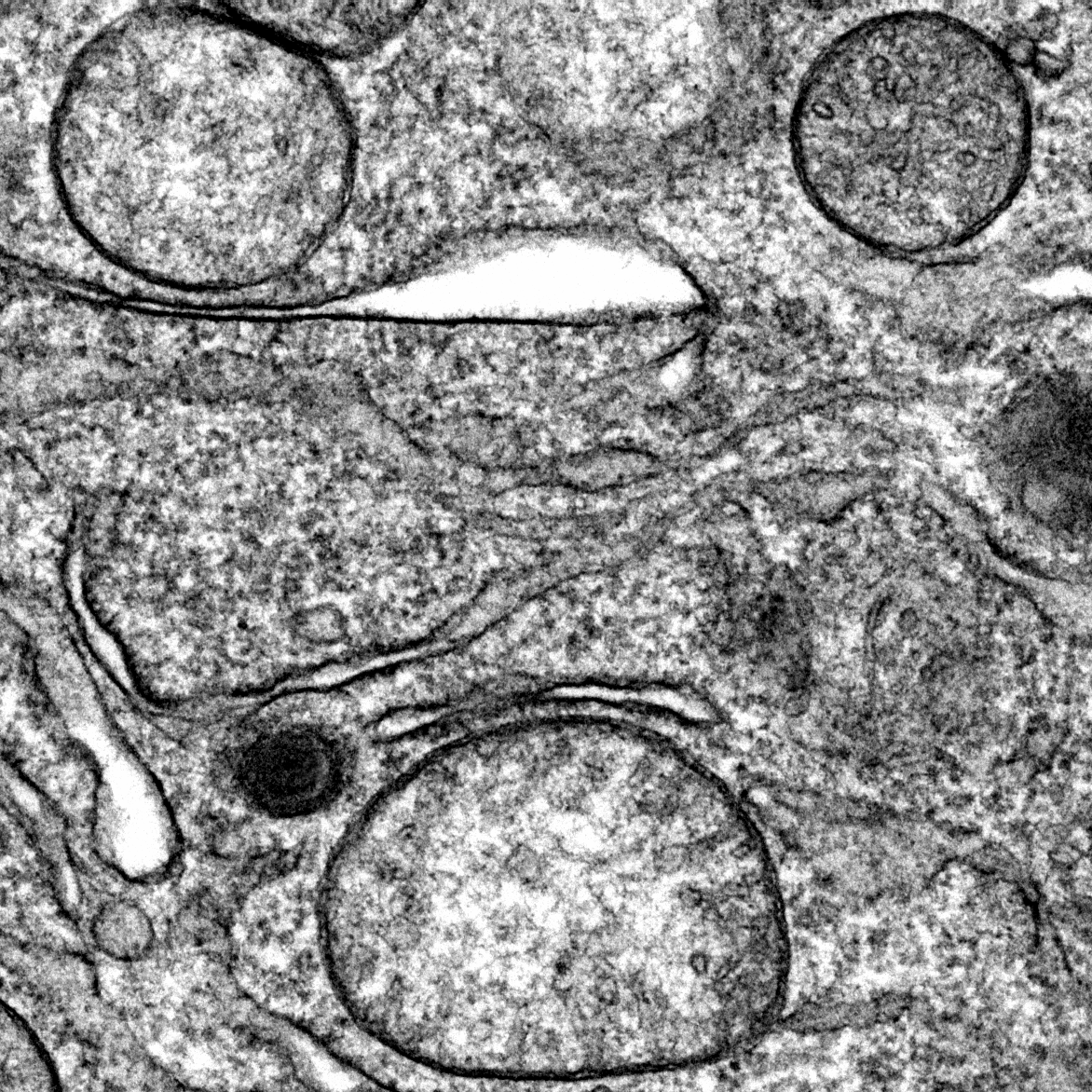

Supplement: Supplementary file 3 — Source data Fig. 2 [file 44319_2024_209_MOESM3_ESM.zip › Figure 2/2B/Control.tif]

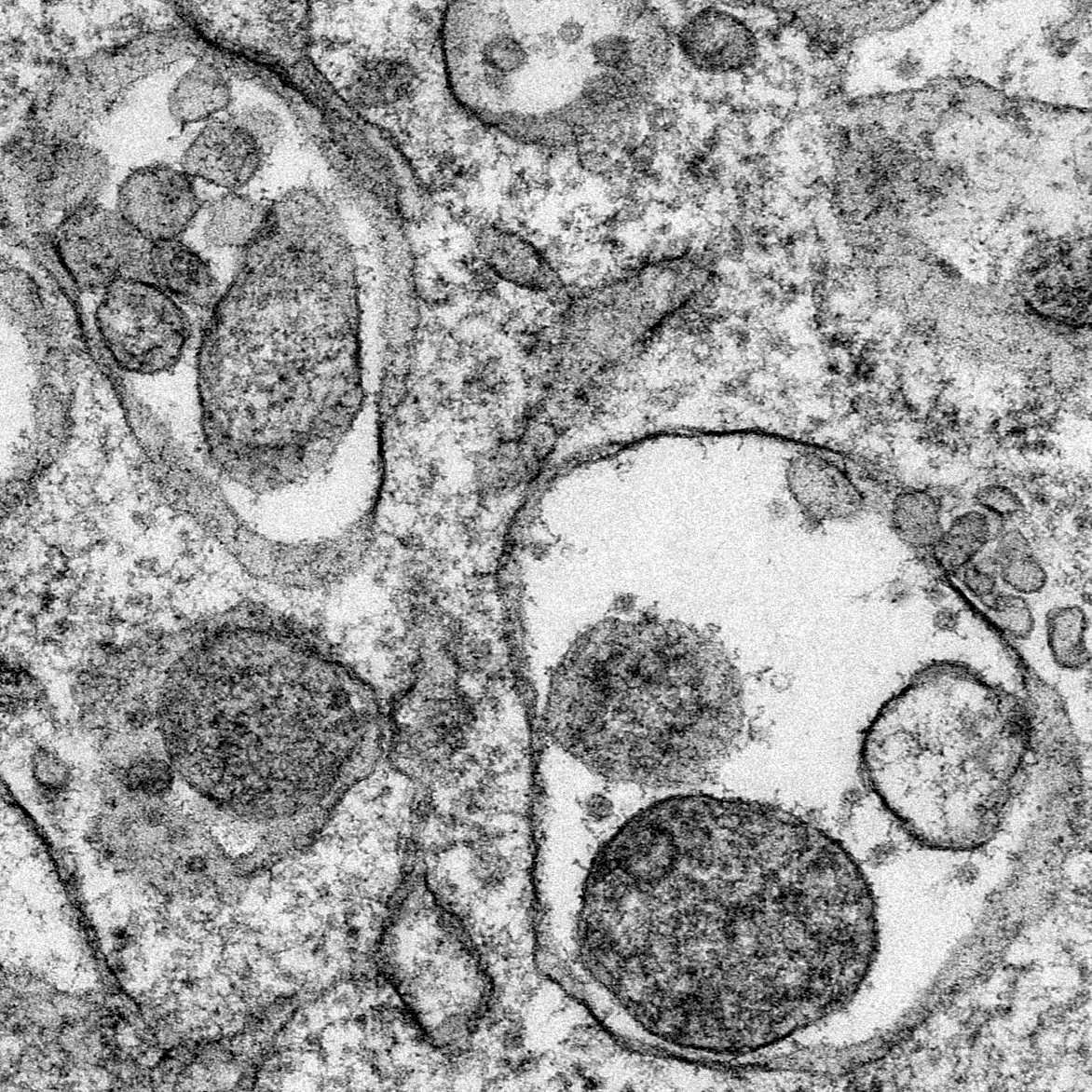

Supplement: Supplementary file 3 — Source data Fig. 2 [file 44319_2024_209_MOESM3_ESM.zip › Figure 2/2B/PFN1 KO.tif]

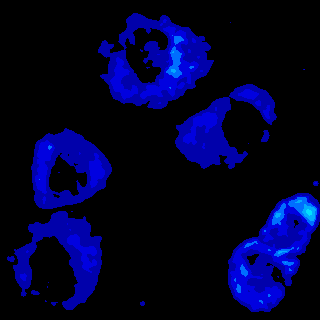

Supplement: Supplementary file 4 — Source data Fig. 3 [file 44319_2024_209_MOESM4_ESM.zip › Figure 3/3G/PFN1 KO TMRE.tif]

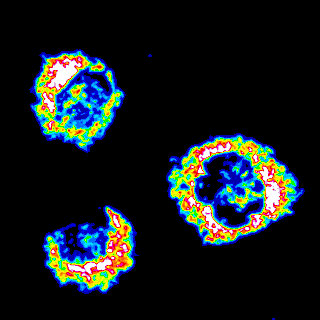

Supplement: Supplementary file 4 — Source data Fig. 3 [file 44319_2024_209_MOESM4_ESM.zip › Figure 3/3G/Control TMRE.tif]

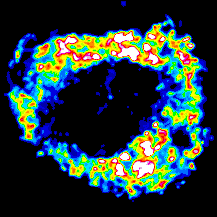

Supplement: Supplementary file 4 — Source data Fig. 3 [file 44319_2024_209_MOESM4_ESM.zip › Figure 3/3I/PFN1 KO MitoSox.tif]

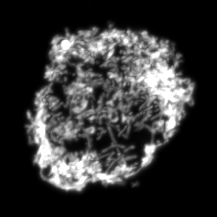

Supplement: Supplementary file 4 — Source data Fig. 3 [file 44319_2024_209_MOESM4_ESM.zip › Figure 3/3I/Control 4xmts-mScarlet.tif]

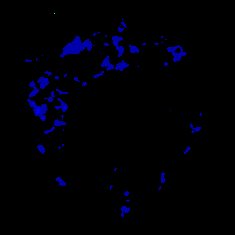

Supplement: Supplementary file 4 — Source data Fig. 3 [file 44319_2024_209_MOESM4_ESM.zip › Figure 3/3I/PFN1 KO + SOD MitoSox.tif]

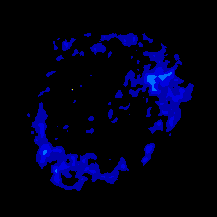

Supplement: Supplementary file 4 — Source data Fig. 3 [file 44319_2024_209_MOESM4_ESM.zip › Figure 3/3I/Control MitoSox.tif]

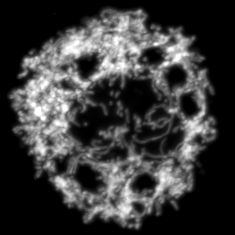

Supplement: Supplementary file 4 — Source data Fig. 3 [file 44319_2024_209_MOESM4_ESM.zip › Figure 3/3I/PFN1 KO + SOD 4xmts-mScarlet.tif]

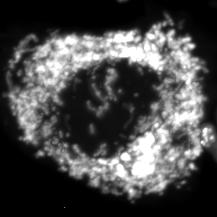

Supplement: Supplementary file 4 — Source data Fig. 3 [file 44319_2024_209_MOESM4_ESM.zip › Figure 3/3I/PFN1 KO 4xmts-mScarlet.tif]

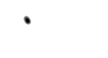

Supplement: Supplementary file 5 — Source data Fig. 4 [file 44319_2024_209_MOESM5_ESM.zip › Figure 4/4E/TOM20 inset.tif]

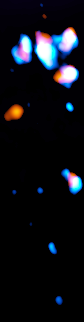

Supplement: Supplementary file 5 — Source data Fig. 4 [file 44319_2024_209_MOESM5_ESM.zip › Figure 4/4E/TOM20 positive MDVs inset.tif]

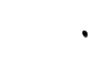

Supplement: Supplementary file 5 — Source data Fig. 4 [file 44319_2024_209_MOESM5_ESM.zip › Figure 4/4E/PDH inset.tif]

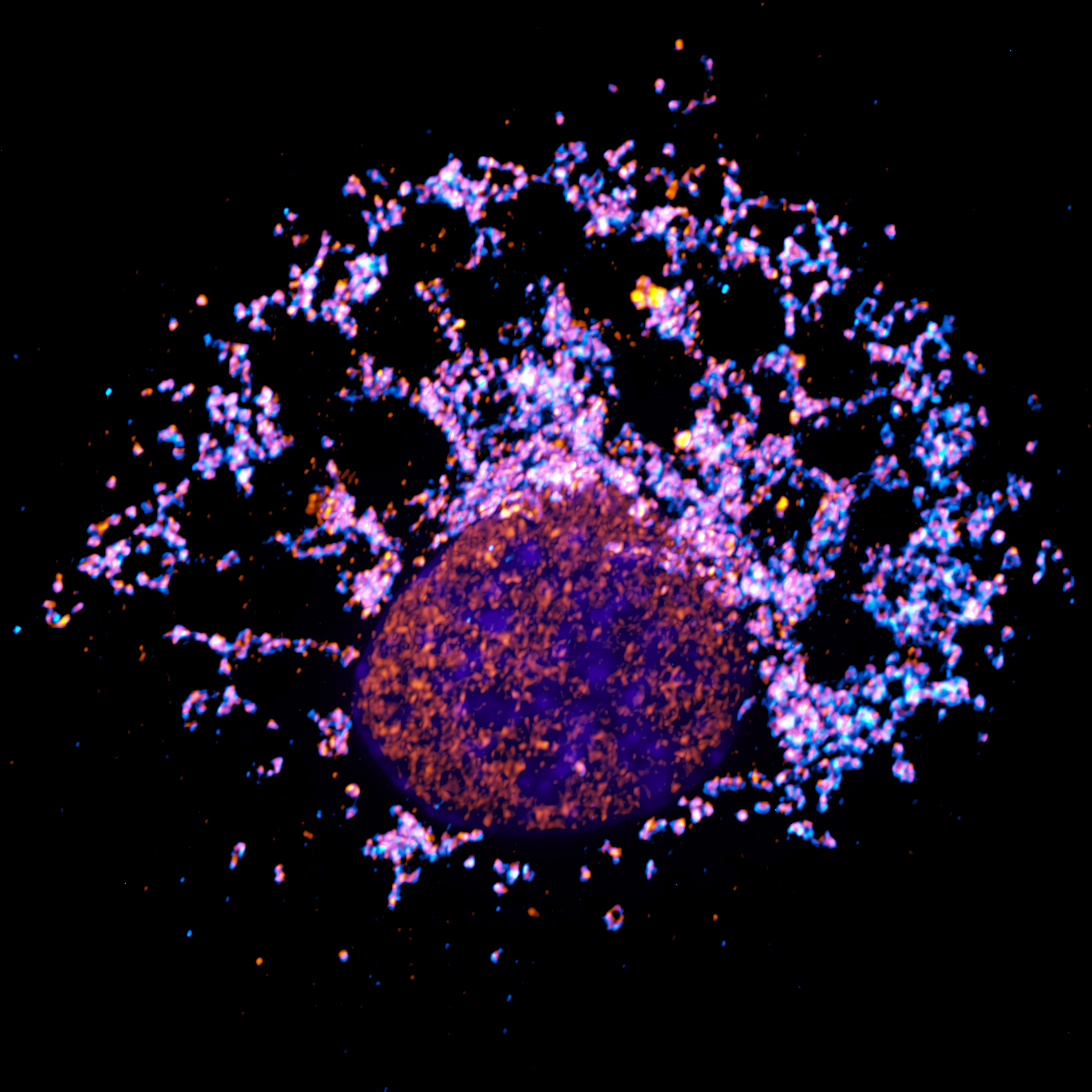

Supplement: Supplementary file 5 — Source data Fig. 4 [file 44319_2024_209_MOESM5_ESM.zip › Figure 4/4E/PFN1 KO TOM20 PDH DAPI.tif]

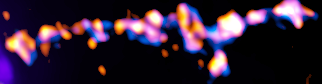

Supplement: Supplementary file 5 — Source data Fig. 4 [file 44319_2024_209_MOESM5_ESM.zip › Figure 4/4E/PDH positive MDVs inset.tif]

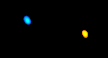

Supplement: Supplementary file 5 — Source data Fig. 4 [file 44319_2024_209_MOESM5_ESM.zip › Figure 4/4E/TOM20 PDH inset merge.tif]

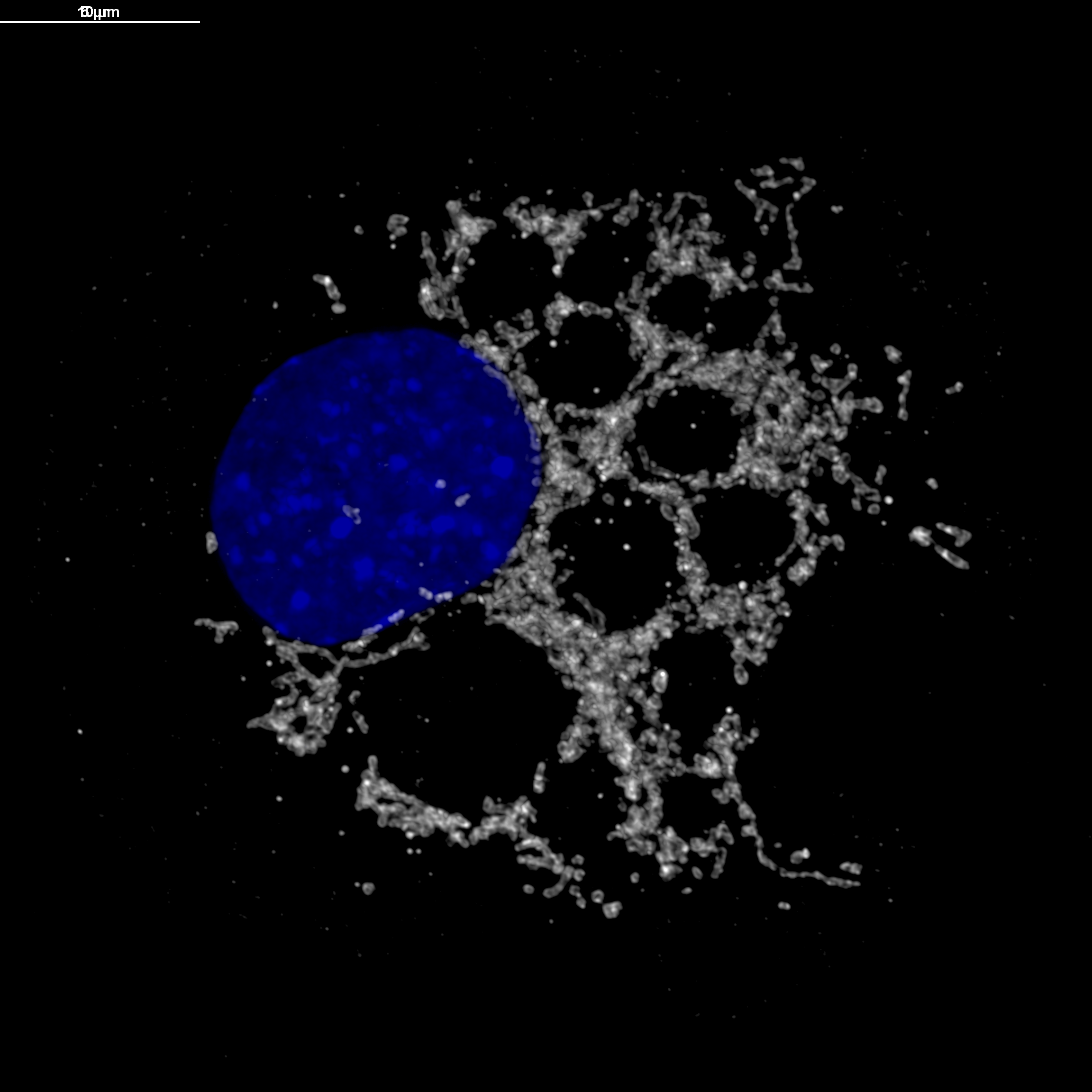

Supplement: Supplementary file 5 — Source data Fig. 4 [file 44319_2024_209_MOESM5_ESM.zip › Figure 4/4B/PFN1 KO Tom20.tif]

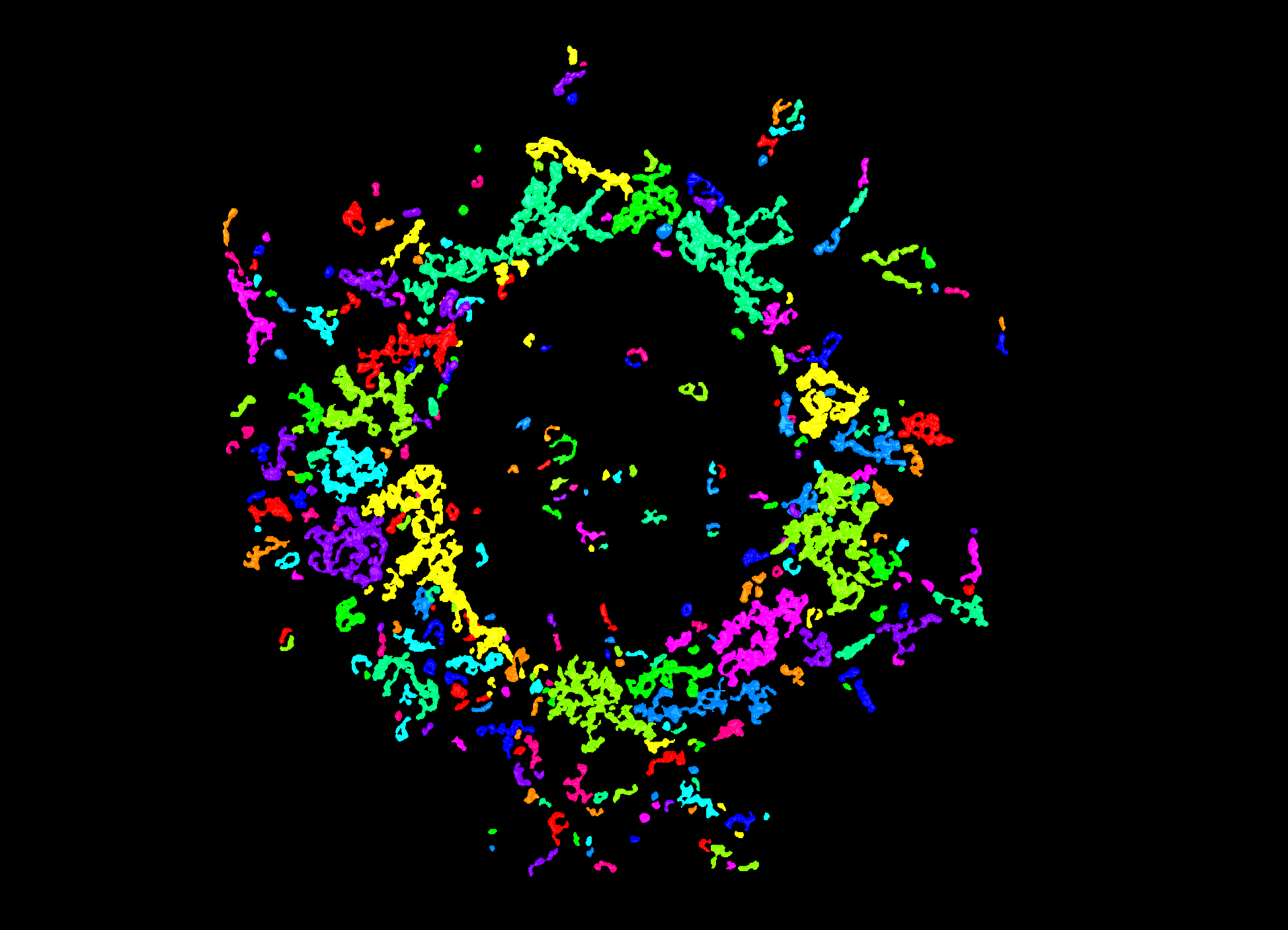

Supplement: Supplementary file 5 — Source data Fig. 4 [file 44319_2024_209_MOESM5_ESM.zip › Figure 4/4B/Control TOM20 segmented.tif]

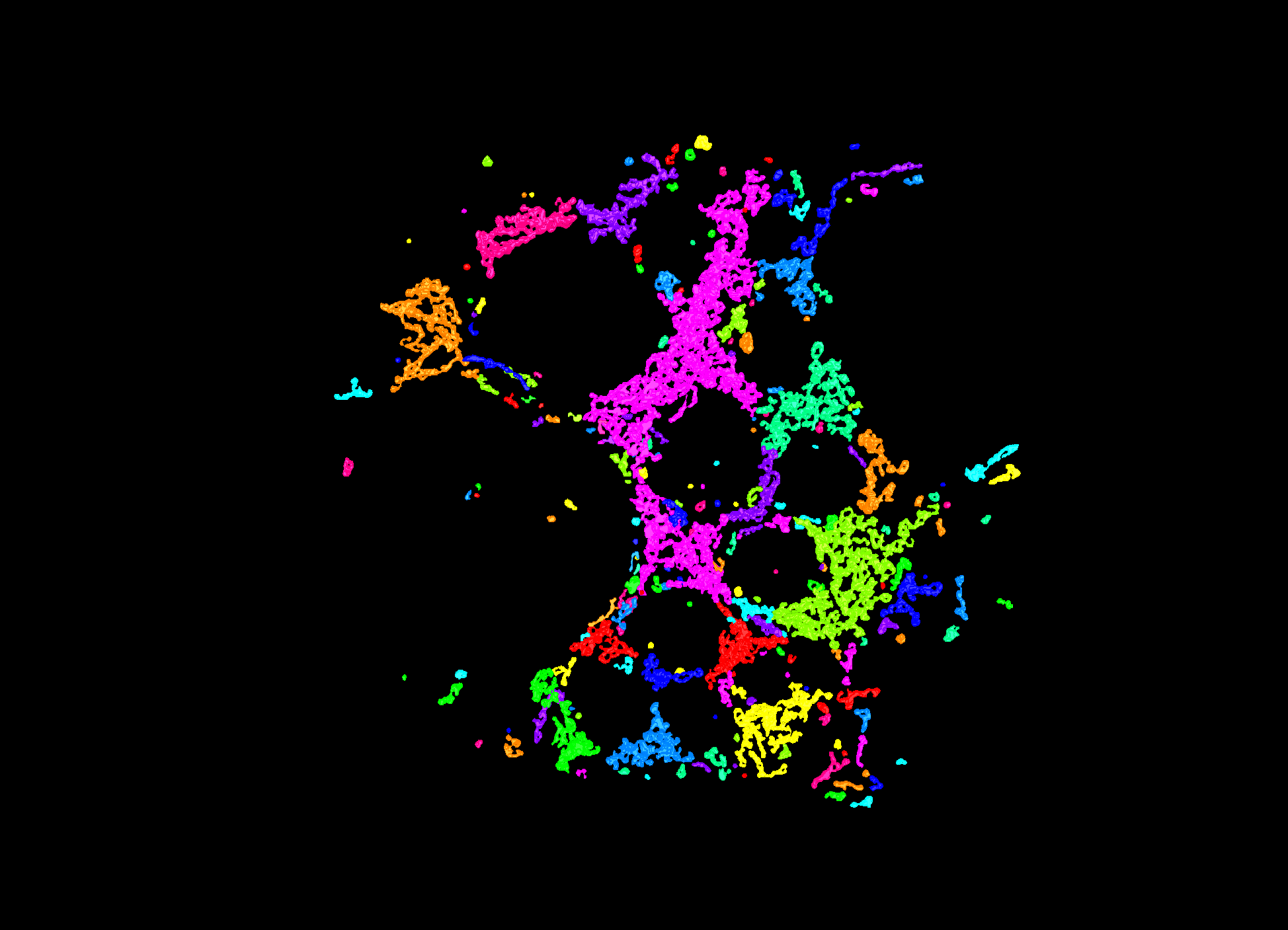

Supplement: Supplementary file 5 — Source data Fig. 4 [file 44319_2024_209_MOESM5_ESM.zip › Figure 4/4B/PFN1 KO TOM20 segmented.tif]

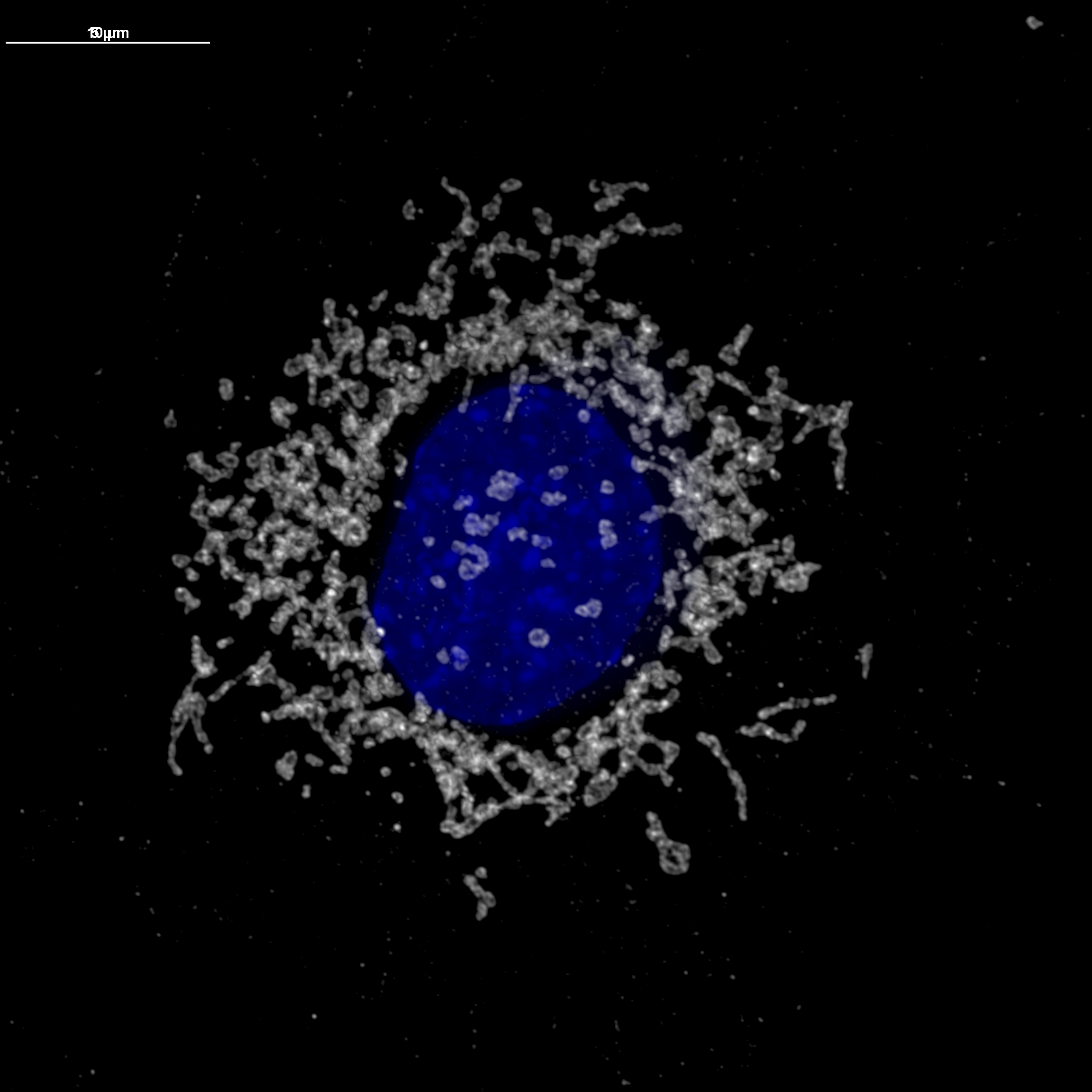

Supplement: Supplementary file 5 — Source data Fig. 4 [file 44319_2024_209_MOESM5_ESM.zip › Figure 4/4B/Control TOM20.tif]

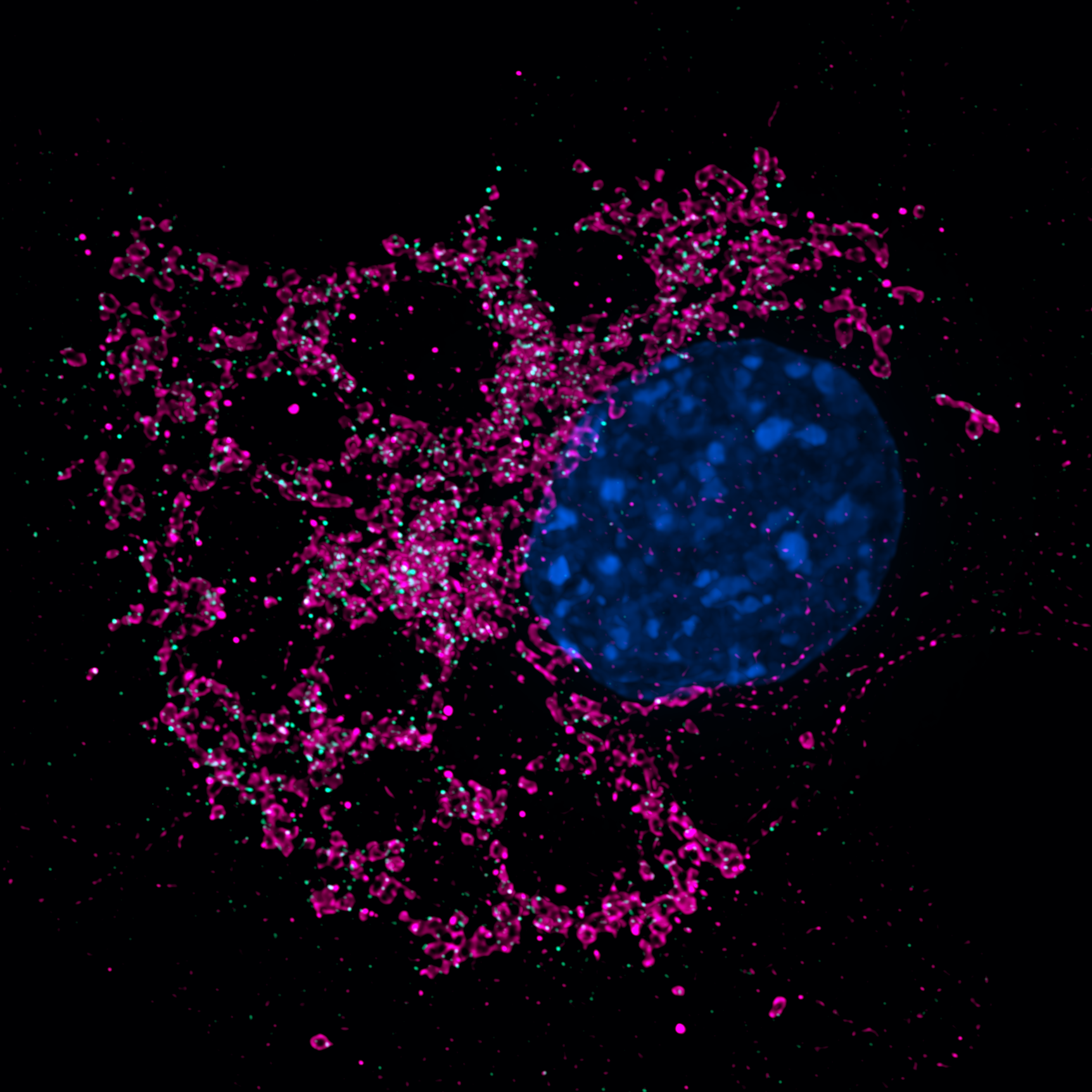

Supplement: Supplementary file 5 — Source data Fig. 4 [file 44319_2024_209_MOESM5_ESM.zip › Figure 4/4F/PFN1 KO TOM20 DRP1 DAPI.tif]

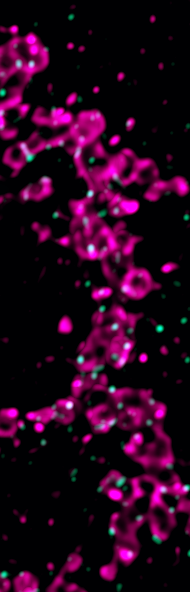

Supplement: Supplementary file 5 — Source data Fig. 4 [file 44319_2024_209_MOESM5_ESM.zip › Figure 4/4F/PFN1 KO TOM20 DRP1 DAPI inset.tif]

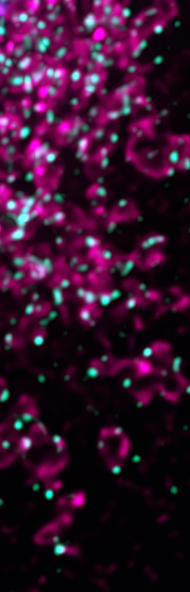

Supplement: Supplementary file 5 — Source data Fig. 4 [file 44319_2024_209_MOESM5_ESM.zip › Figure 4/4F/Control TOM20 DRP1 DAPI inset.tif]

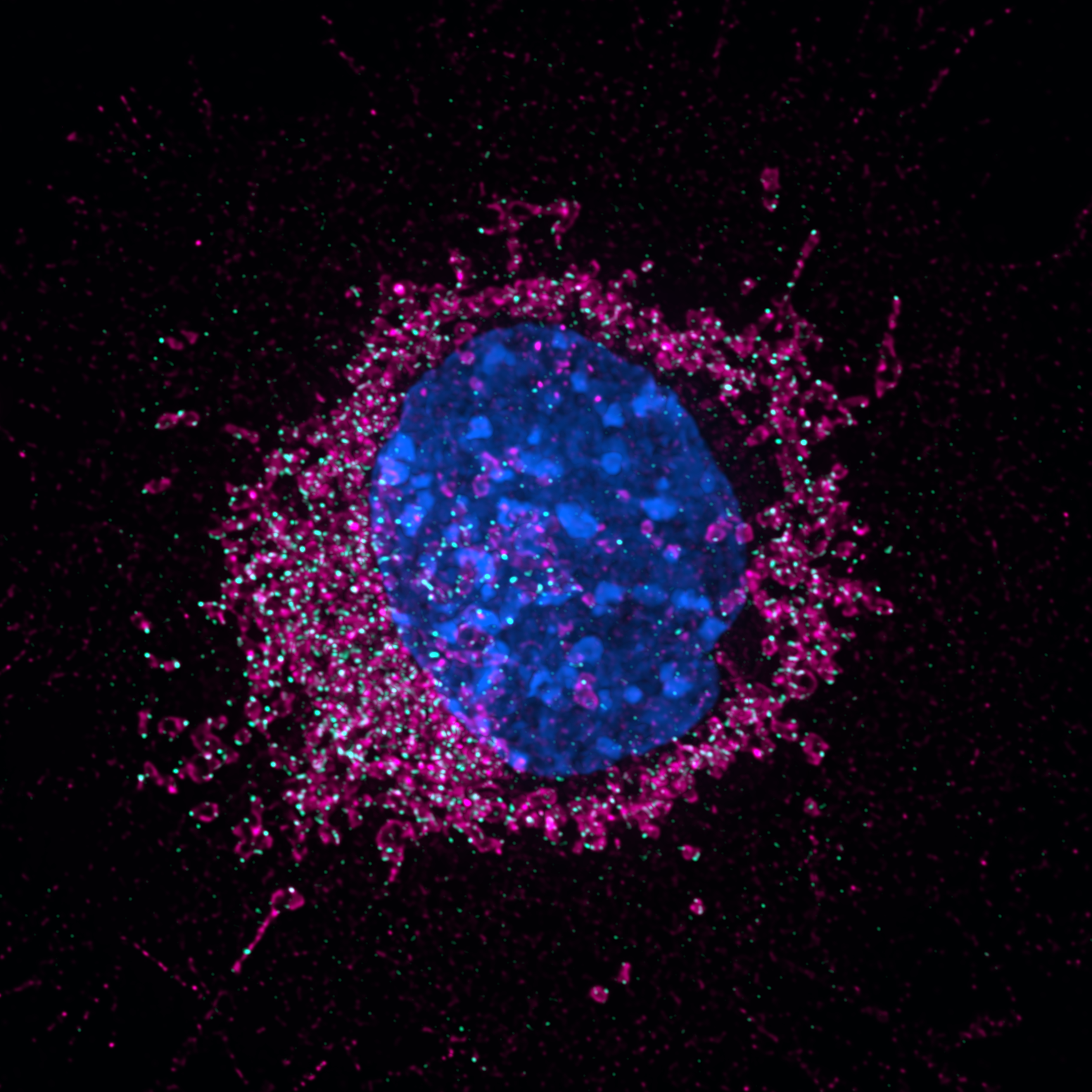

Supplement: Supplementary file 5 — Source data Fig. 4 [file 44319_2024_209_MOESM5_ESM.zip › Figure 4/4F/Control TOM20 DRP1 DAPI.tif]

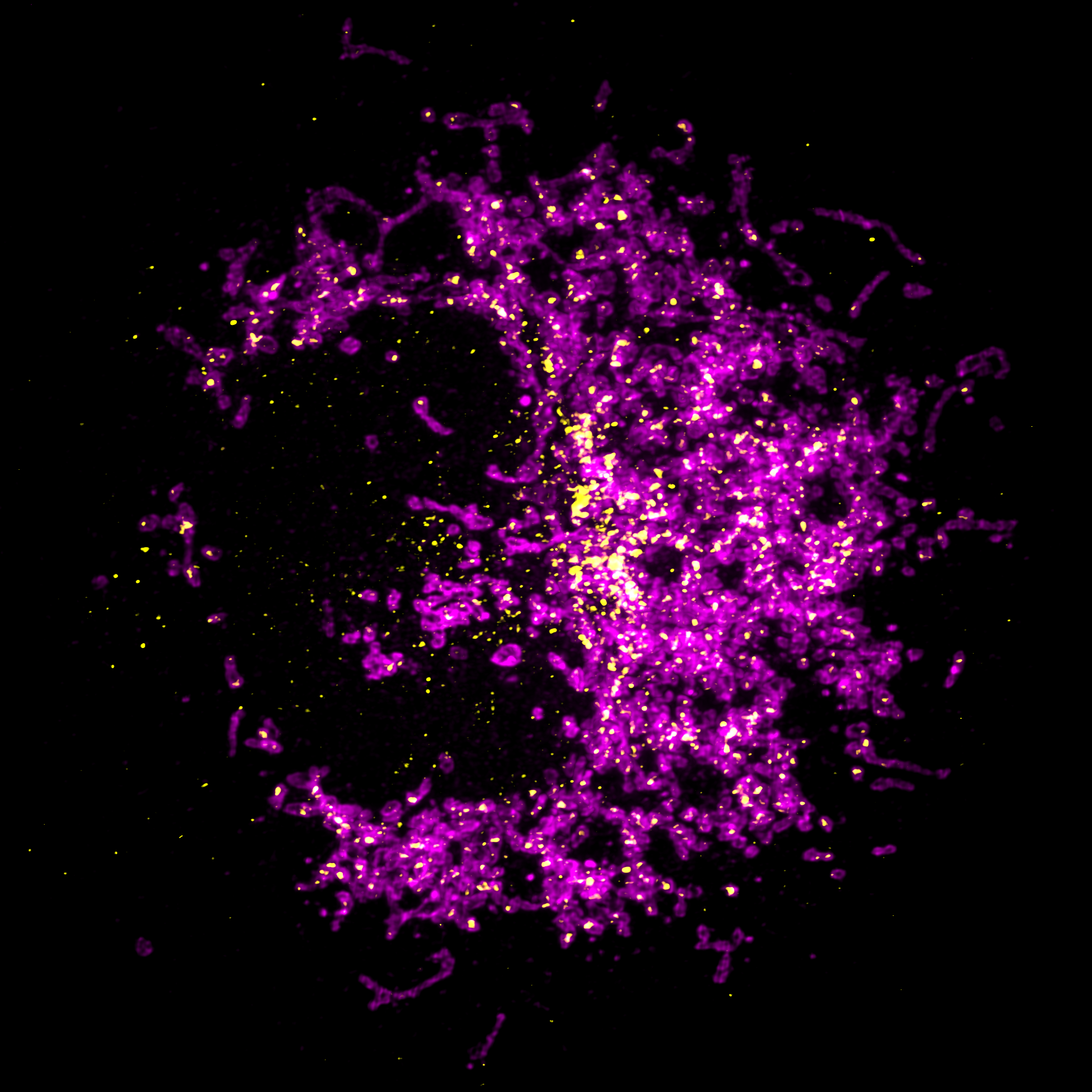

Supplement: Supplementary file 5 — Source data Fig. 4 [file 44319_2024_209_MOESM5_ESM.zip › Figure 4/4G/PFN1 KO TOM20 MFN2 DAPI.tif]

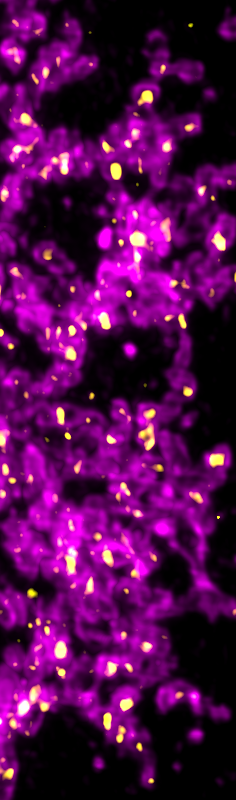

Supplement: Supplementary file 5 — Source data Fig. 4 [file 44319_2024_209_MOESM5_ESM.zip › Figure 4/4G/PFN1 KO TOM20 DRP1 DAPI inset.tif]

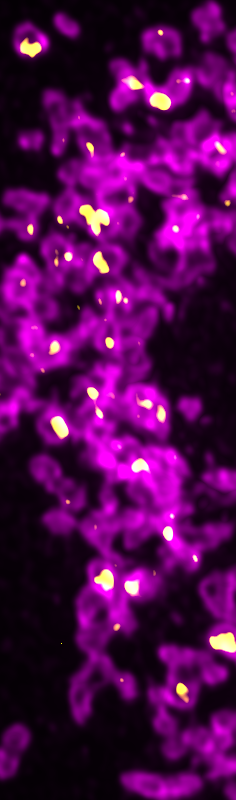

Supplement: Supplementary file 5 — Source data Fig. 4 [file 44319_2024_209_MOESM5_ESM.zip › Figure 4/4G/Control TOM20 DRP1 DAPI inset.tif]

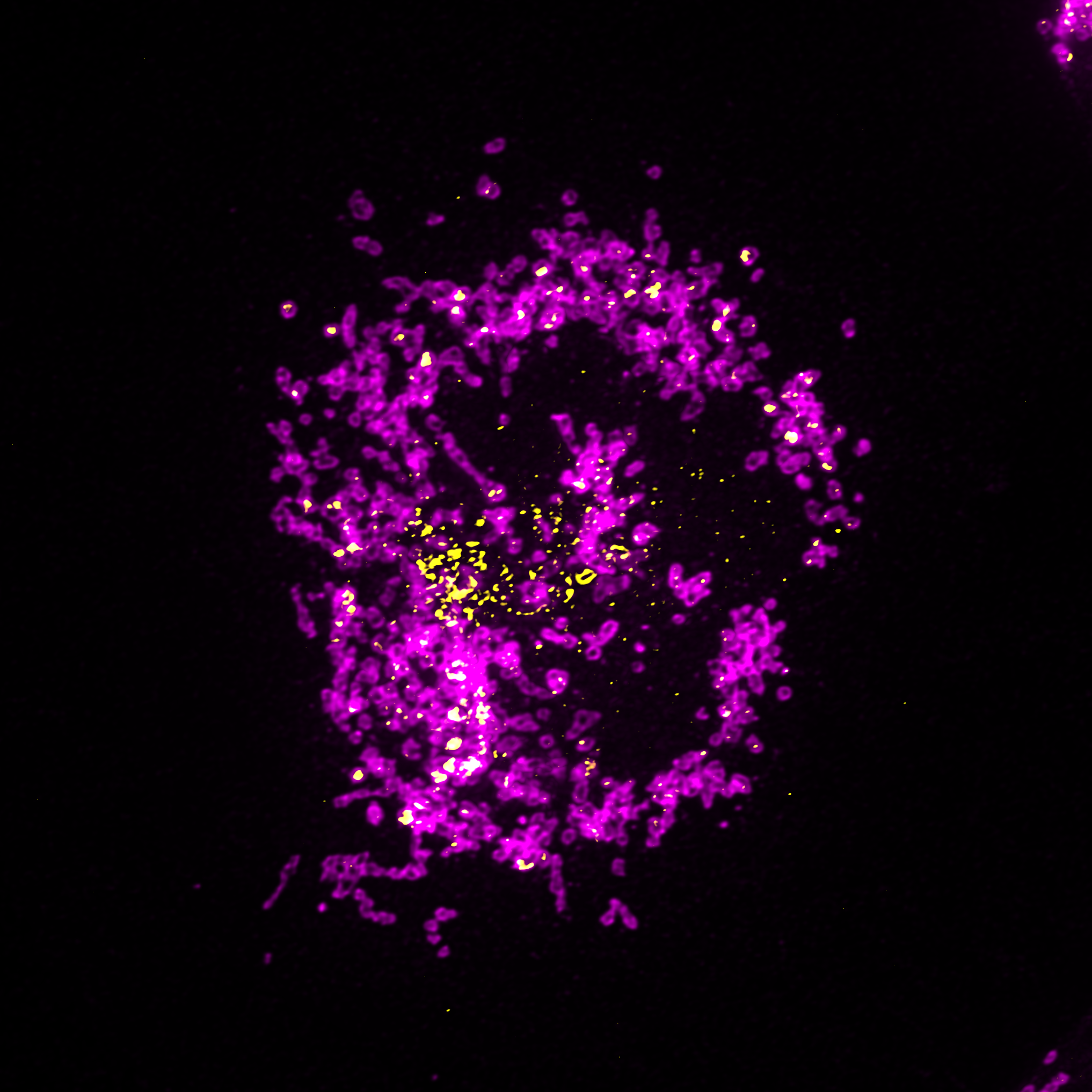

Supplement: Supplementary file 5 — Source data Fig. 4 [file 44319_2024_209_MOESM5_ESM.zip › Figure 4/4G/Control TOM20 DRP1 DAPI.tif]

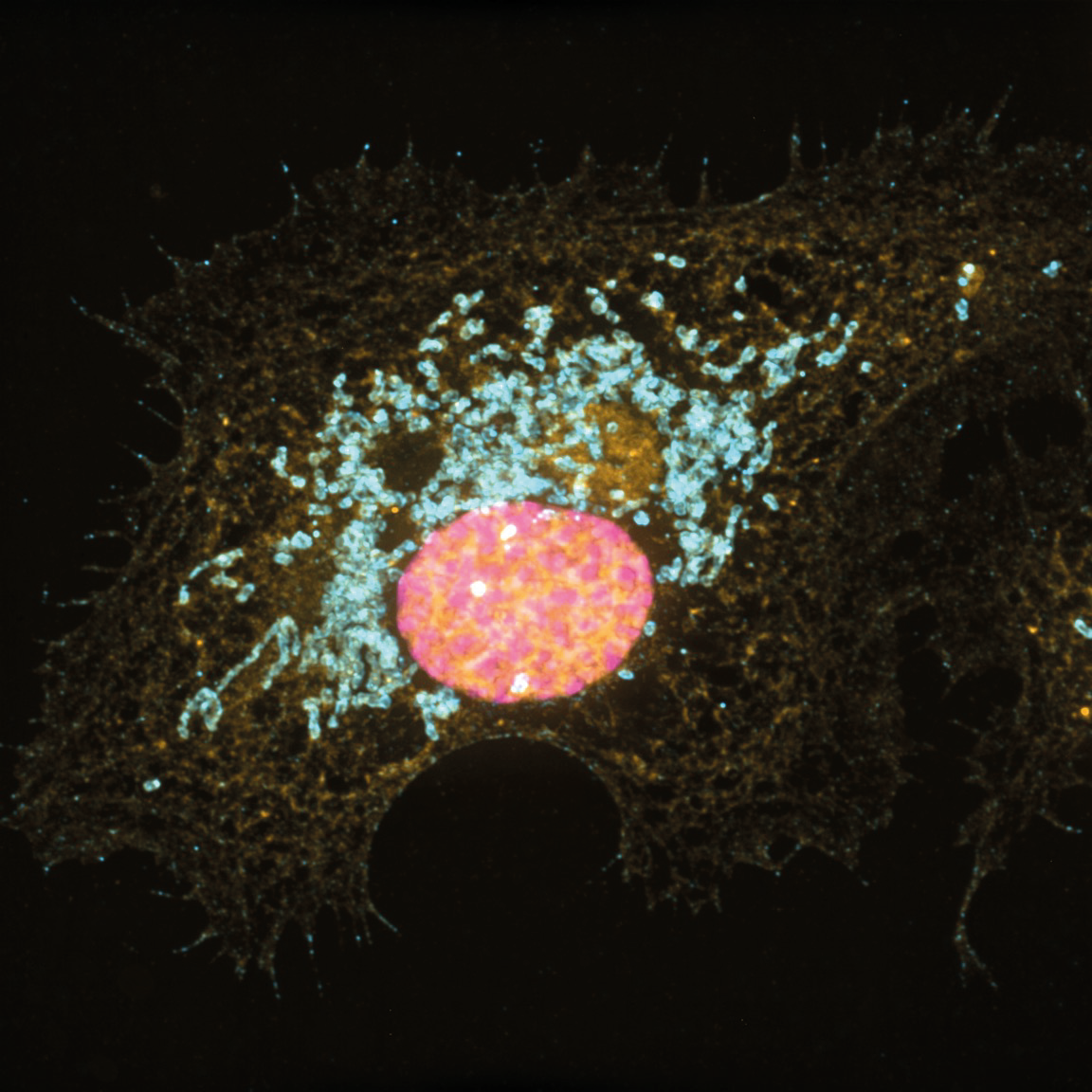

Supplement: Supplementary file 6 — Source data Fig. 5 [file 44319_2024_209_MOESM6_ESM.zip › Figure 5/5A/TOM20 GFP-PFN1 DAPI merge.tif]

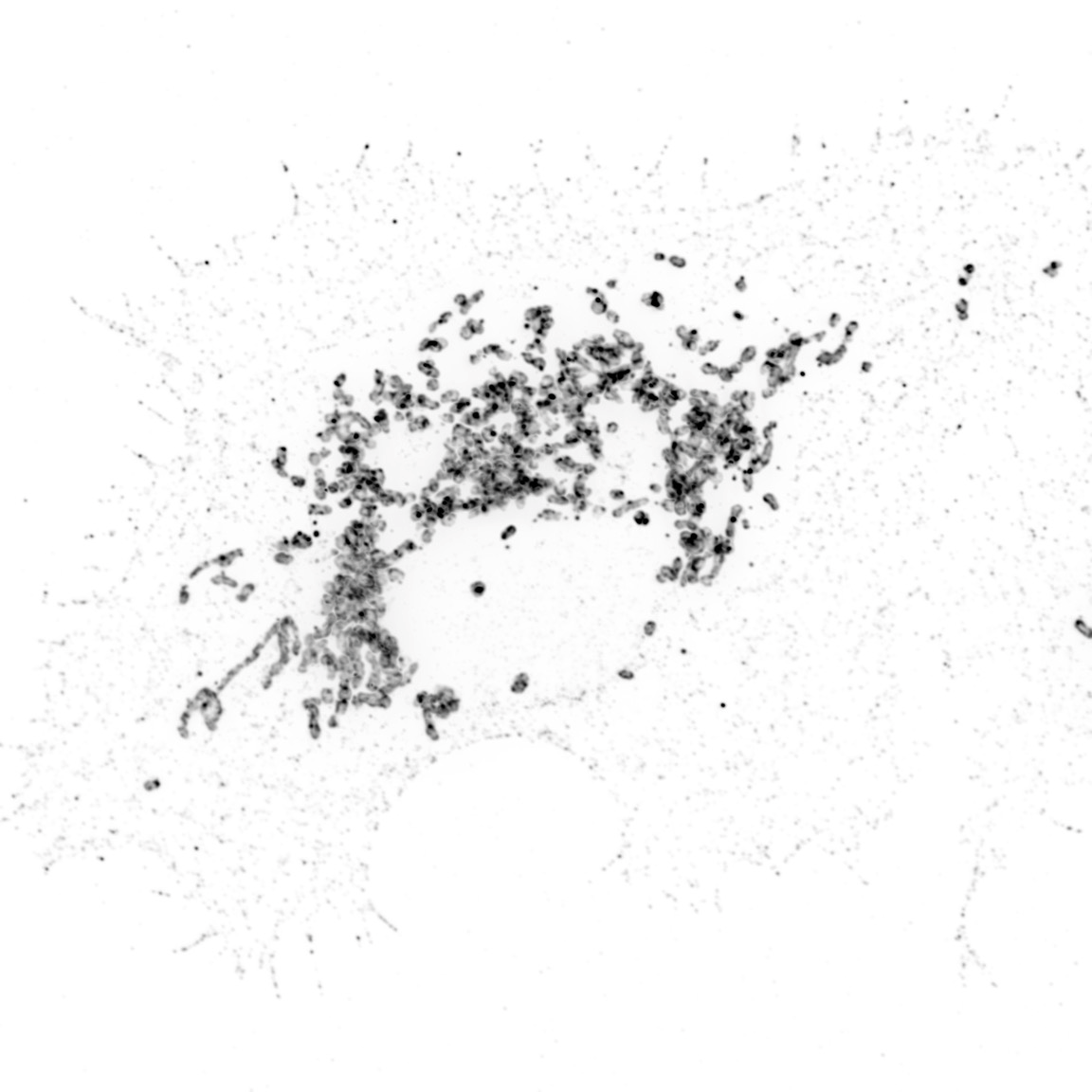

Supplement: Supplementary file 6 — Source data Fig. 5 [file 44319_2024_209_MOESM6_ESM.zip › Figure 5/5A/TOM20.tif]

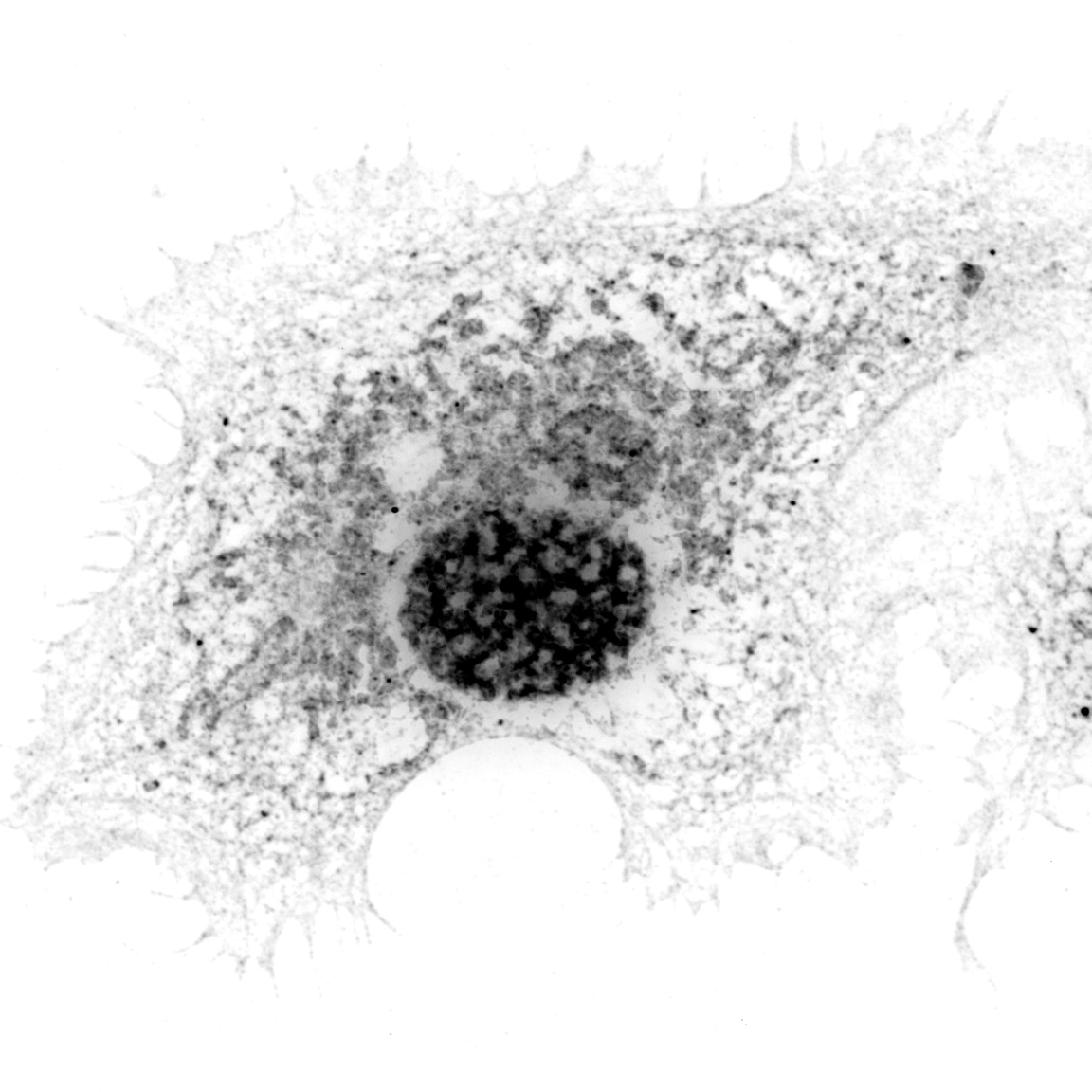

Supplement: Supplementary file 6 — Source data Fig. 5 [file 44319_2024_209_MOESM6_ESM.zip › Figure 5/5A/GFP-PFN1.tif]

Figure 5C

OMM vs. IMM PFN1 localization

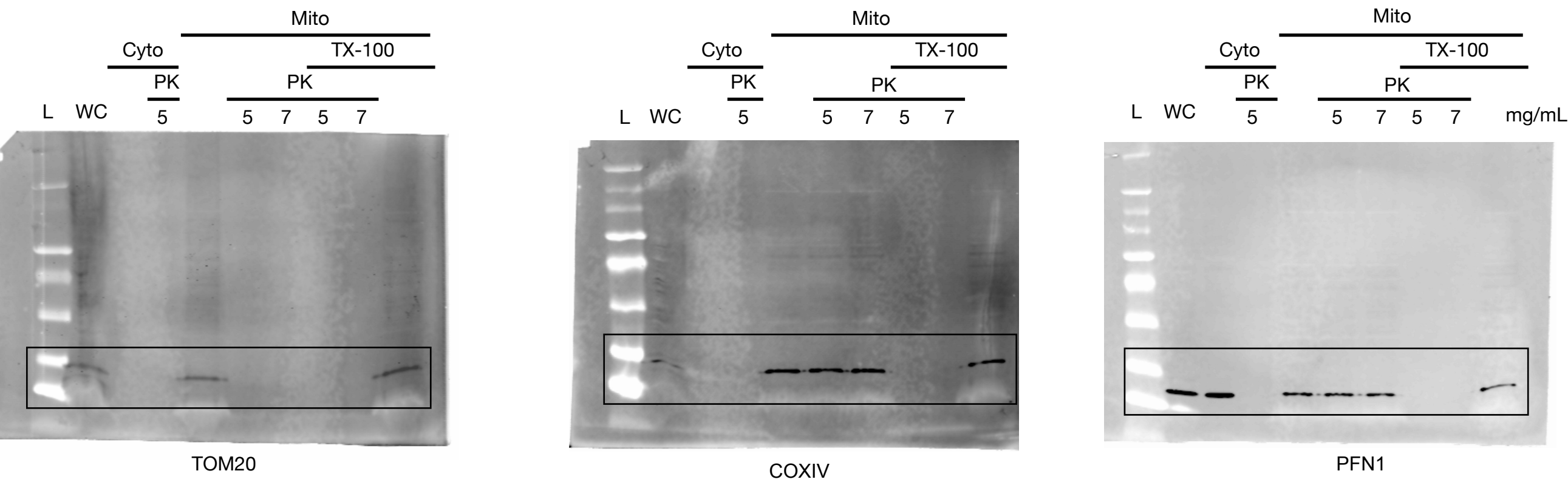

IMS vs Matrix PFN1 localization

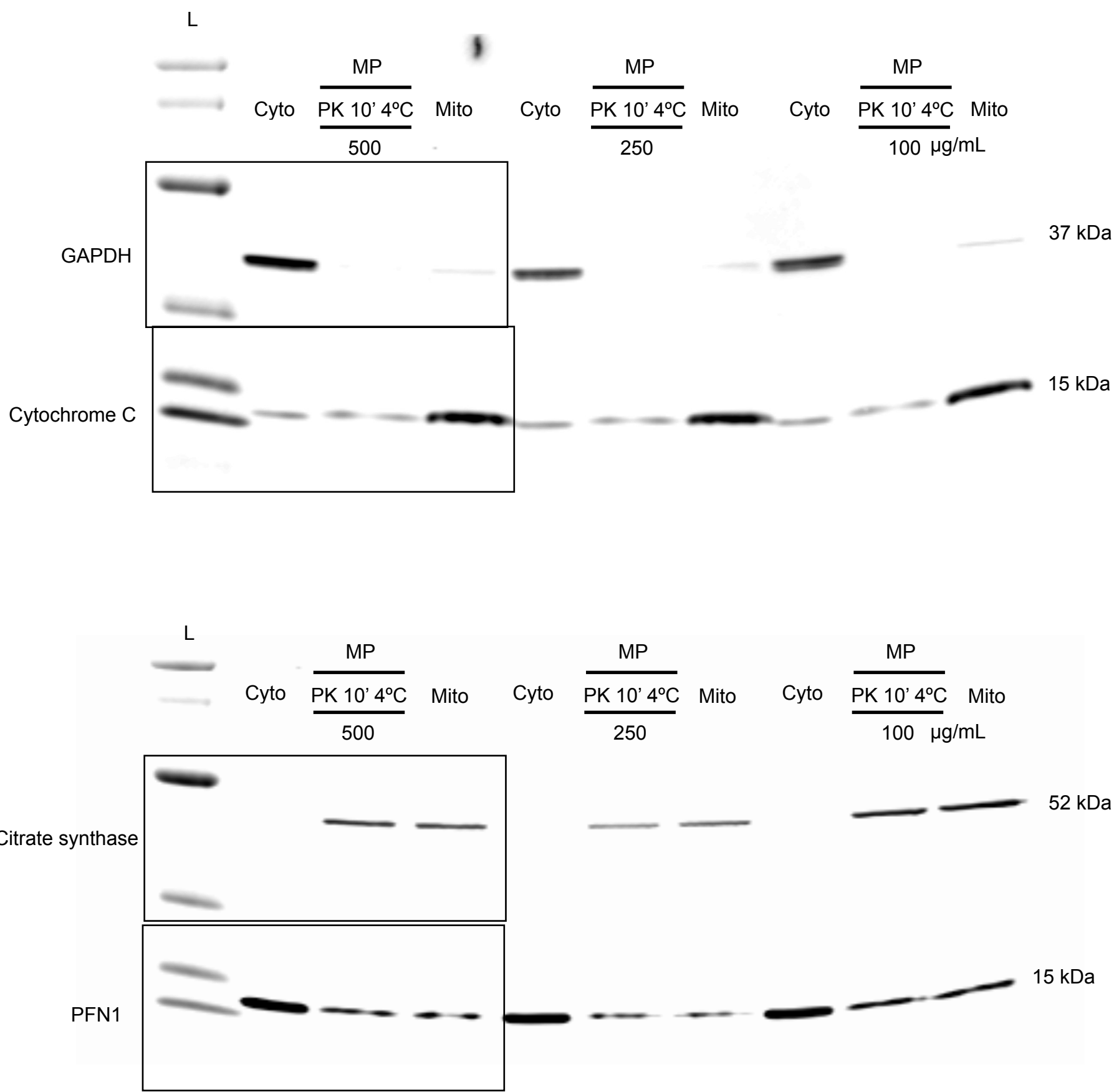

Supplement: Supplementary file 6 — Source data Fig. 5 [file 44319_2024_209_MOESM6_ESM.zip › Figure 5/5C/5C westerns.pdf]

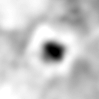

Supplement: Supplementary file 6 — Source data Fig. 5 [file 44319_2024_209_MOESM6_ESM.zip › Figure 5/5D/Inset 3 GFP-PFN1.tif]

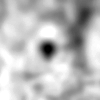

Supplement: Supplementary file 6 — Source data Fig. 5 [file 44319_2024_209_MOESM6_ESM.zip › Figure 5/5D/Inset 2 GFP-PFN1.tif]

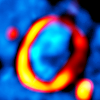

Supplement: Supplementary file 6 — Source data Fig. 5 [file 44319_2024_209_MOESM6_ESM.zip › Figure 5/5D/Inset 1 merge.tif]

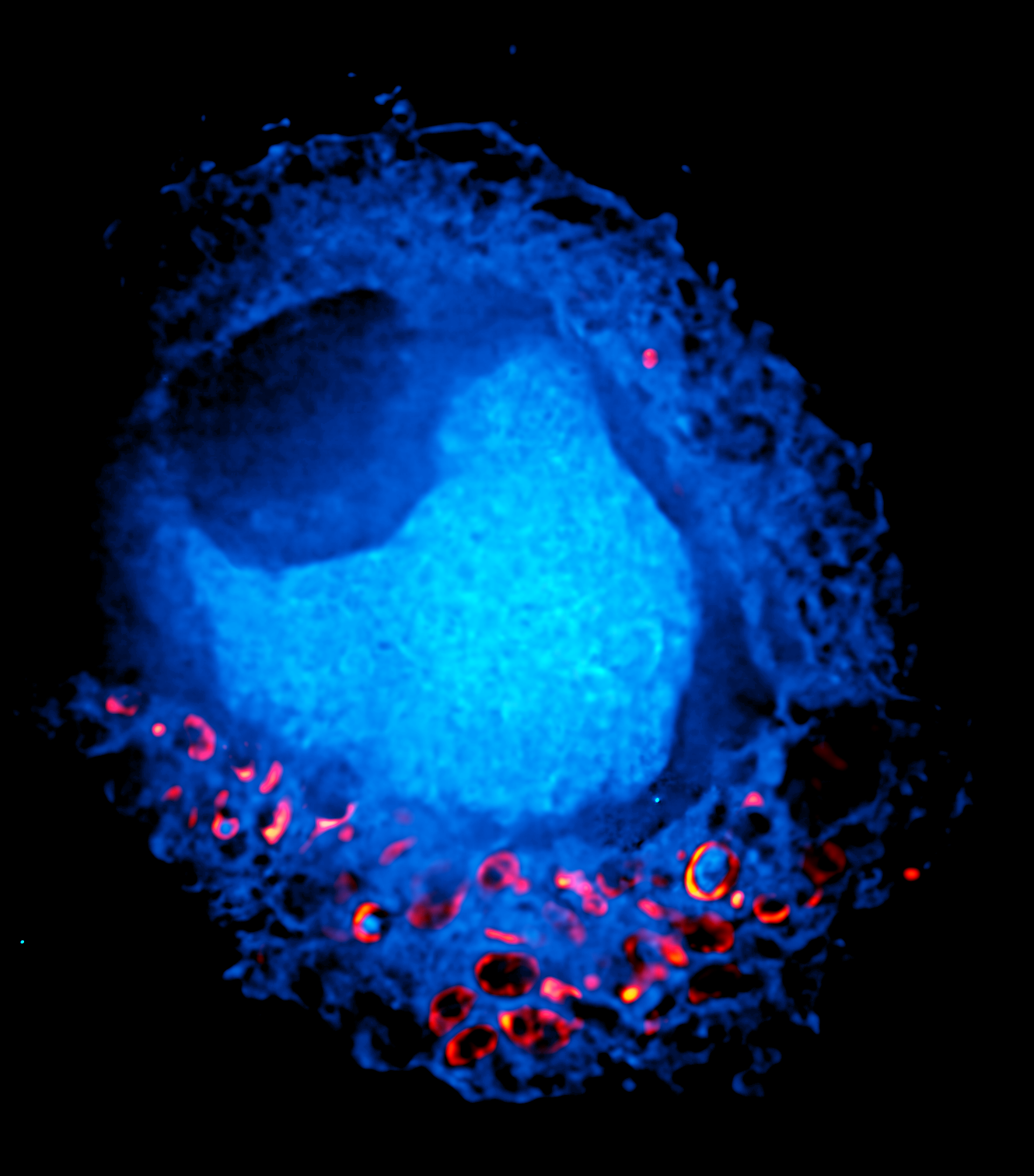

Supplement: Supplementary file 6 — Source data Fig. 5 [file 44319_2024_209_MOESM6_ESM.zip › Figure 5/5D/4xmts-mScarlet GFP-PFN1.tif]

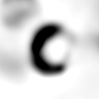

Supplement: Supplementary file 6 — Source data Fig. 5 [file 44319_2024_209_MOESM6_ESM.zip › Figure 5/5D/Inset 3 4xmts-mScarlet.tif]

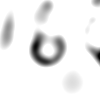

Supplement: Supplementary file 6 — Source data Fig. 5 [file 44319_2024_209_MOESM6_ESM.zip › Figure 5/5D/Inset 2 4xmts-mScarlet.tif]

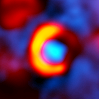

Supplement: Supplementary file 6 — Source data Fig. 5 [file 44319_2024_209_MOESM6_ESM.zip › Figure 5/5D/Inset 3 merge.tif]

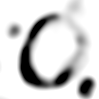

Supplement: Supplementary file 6 — Source data Fig. 5 [file 44319_2024_209_MOESM6_ESM.zip › Figure 5/5D/Inset 1 4xmts-mScarlet.tif]

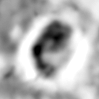

Supplement: Supplementary file 6 — Source data Fig. 5 [file 44319_2024_209_MOESM6_ESM.zip › Figure 5/5D/Inset 1 GFP-PFN1.tif]

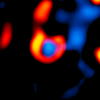

Supplement: Supplementary file 6 — Source data Fig. 5 [file 44319_2024_209_MOESM6_ESM.zip › Figure 5/5D/Inset 2 merge.tif]

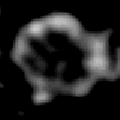

Supplement: Supplementary file 6 — Source data Fig. 5 [file 44319_2024_209_MOESM6_ESM.zip › Figure 5/5B/TOM20 inset.tif]

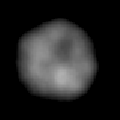

Supplement: Supplementary file 6 — Source data Fig. 5 [file 44319_2024_209_MOESM6_ESM.zip › Figure 5/5B/GFP-PFN1-M114T inset.tif]

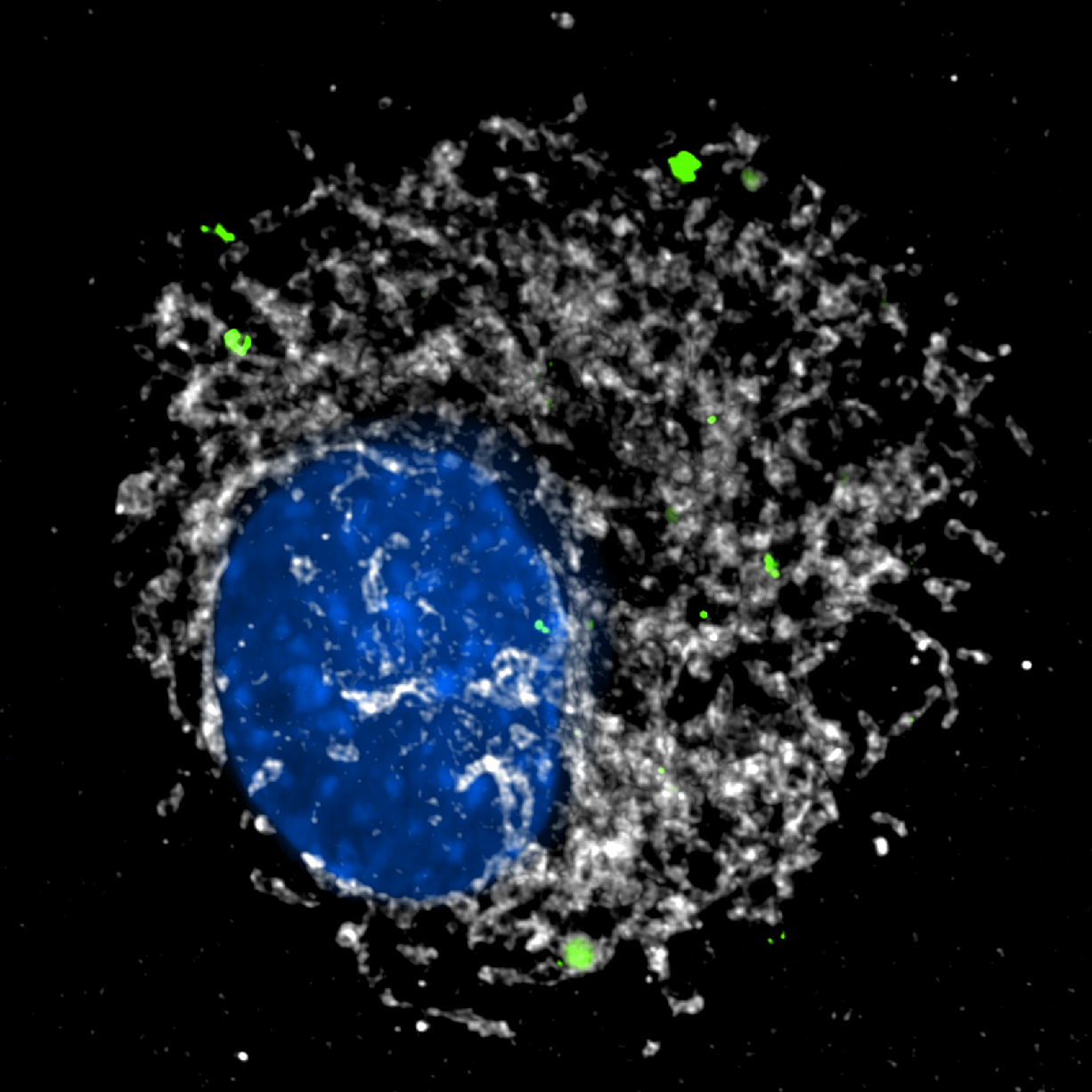

Supplement: Supplementary file 6 — Source data Fig. 5 [file 44319_2024_209_MOESM6_ESM.zip › Figure 5/5B/GFP-PFN1-M114T TOM20 DAPI.tif]

Fig. EV1B

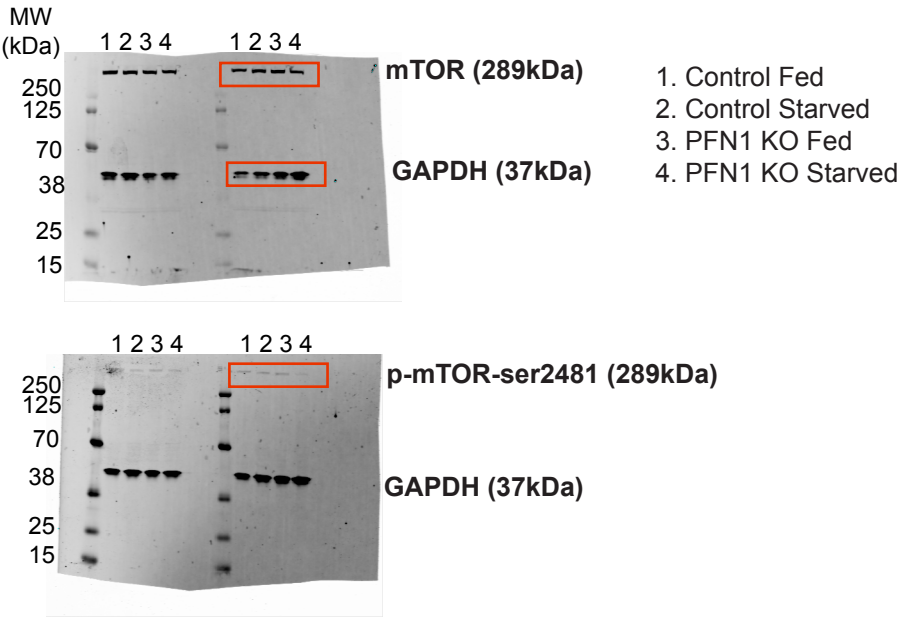

Supplement: Supplementary file 7 — Figure EV1-3 Source Data [file 44319_2024_209_MOESM7_ESM.zip › Figure EV1/EV1B/EV1B westerns.pdf]

Fig. EV1A

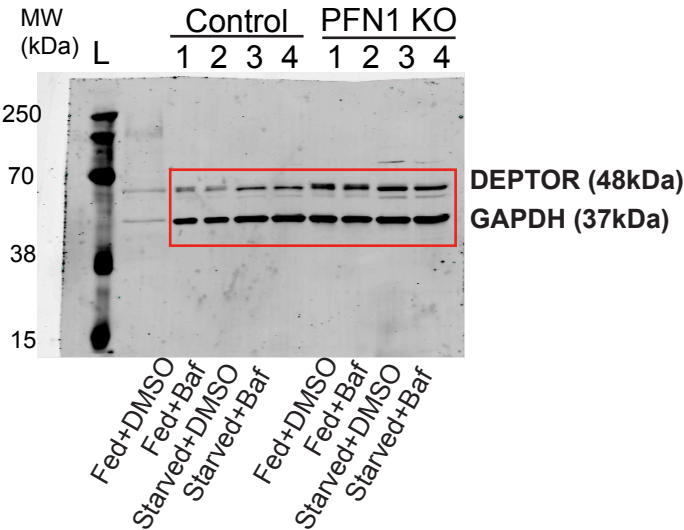

Supplement: Supplementary file 7 — Figure EV1-3 Source Data [file 44319_2024_209_MOESM7_ESM.zip › Figure EV1/EV1A/EV1A western.pdf]

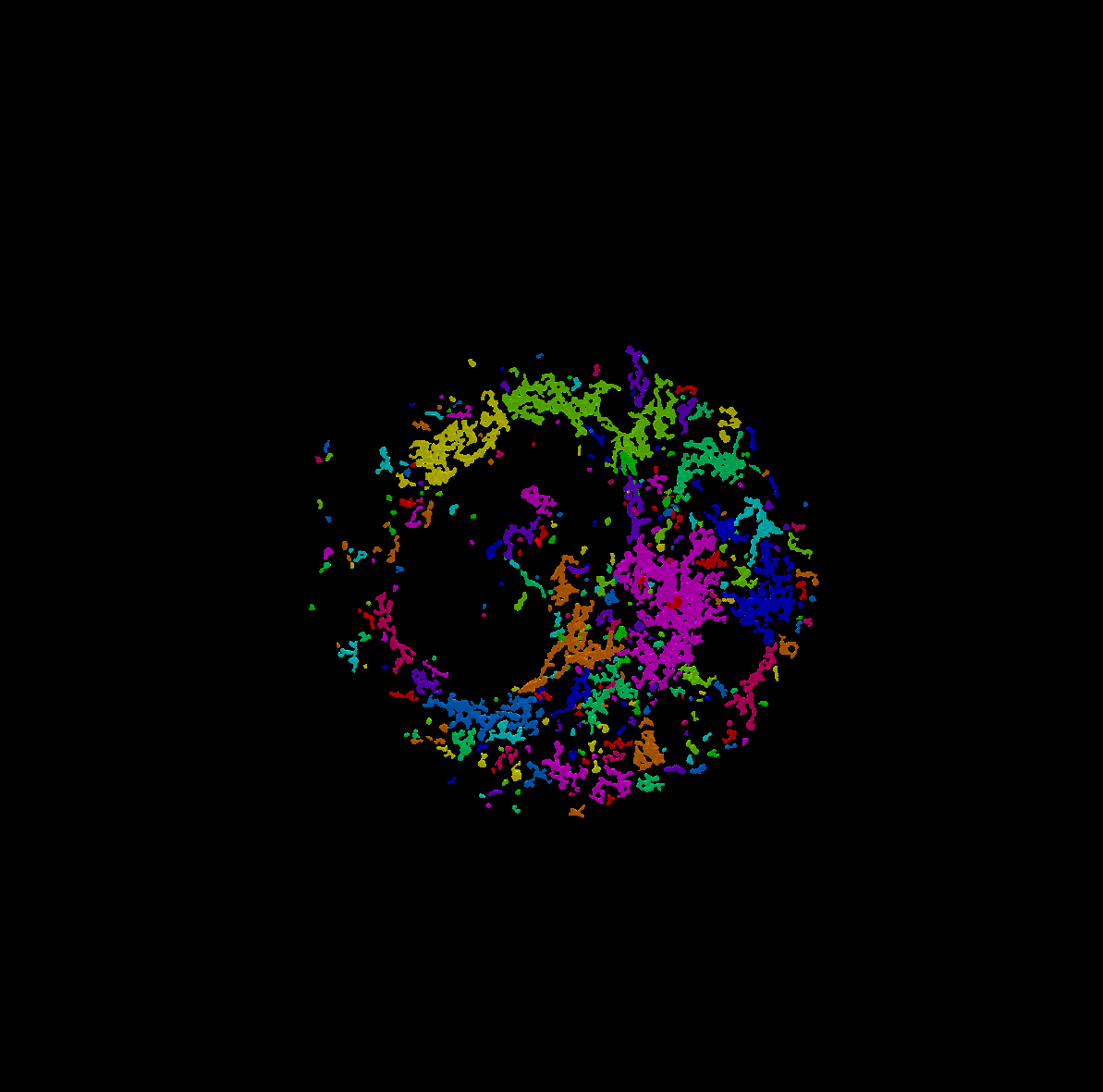

Supplement: Supplementary file 7 — Figure EV1-3 Source Data [file 44319_2024_209_MOESM7_ESM.zip › Figure EV2/EV2C/PFN1 KO + GFP-PFN1-R88E TOM20 segmented.tif]

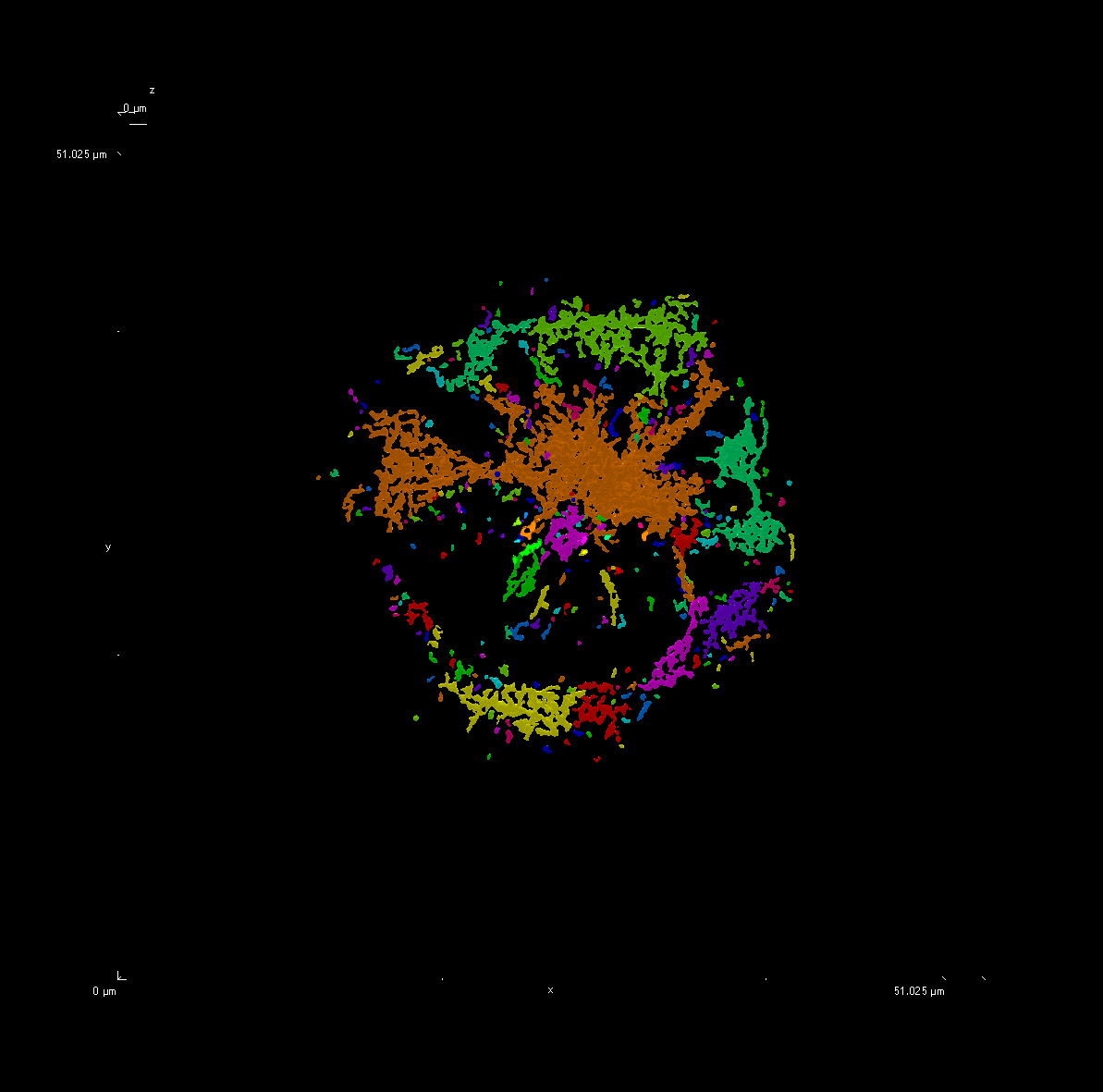

Supplement: Supplementary file 7 — Figure EV1-3 Source Data [file 44319_2024_209_MOESM7_ESM.zip › Figure EV2/EV2C/PFN1 KO + GFP TOM20 segmented.tif]

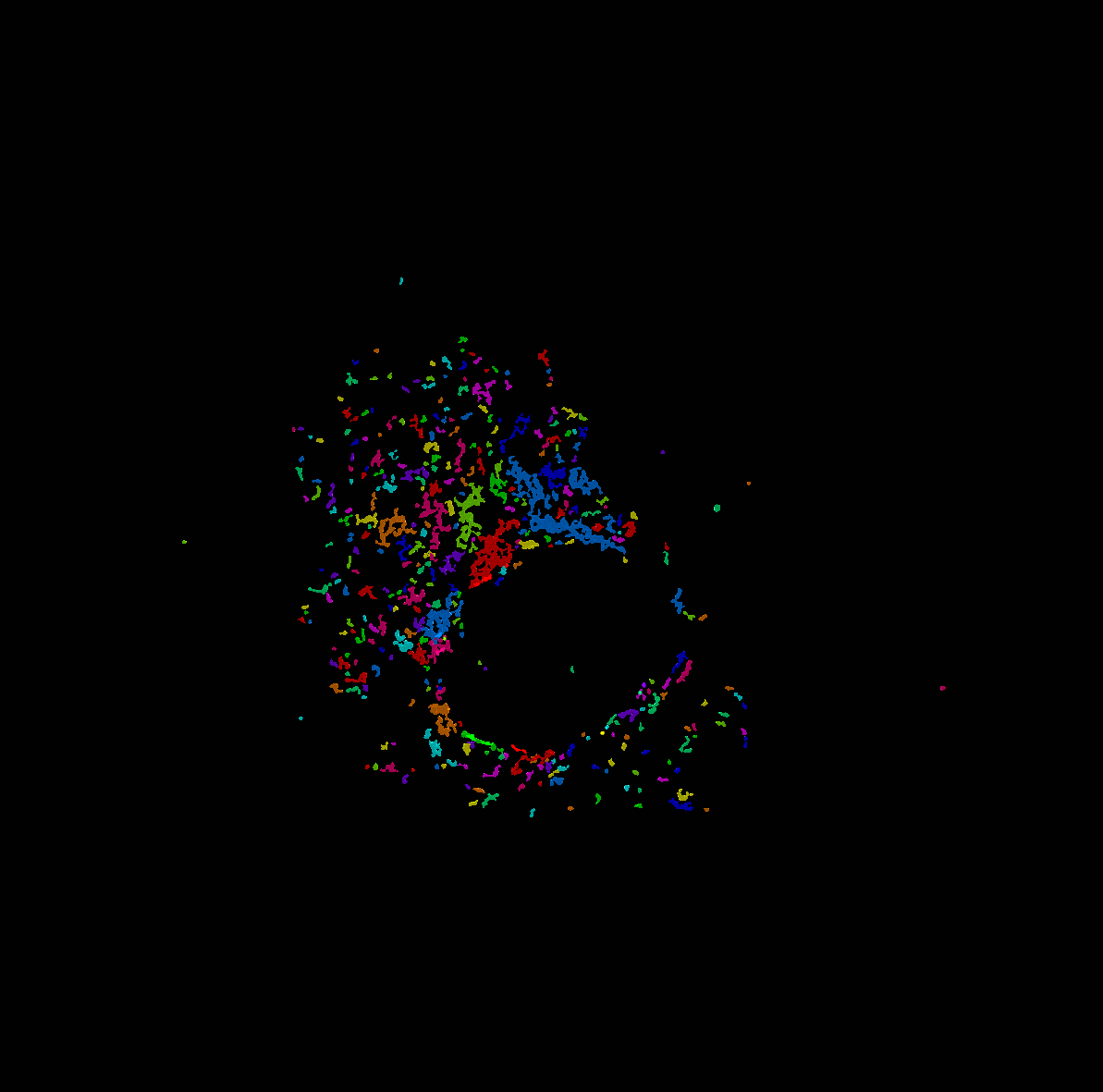

Supplement: Supplementary file 7 — Figure EV1-3 Source Data [file 44319_2024_209_MOESM7_ESM.zip › Figure EV2/EV2C/PFN1 KO + GFP-PFN1 TOM20 segmented.tif]

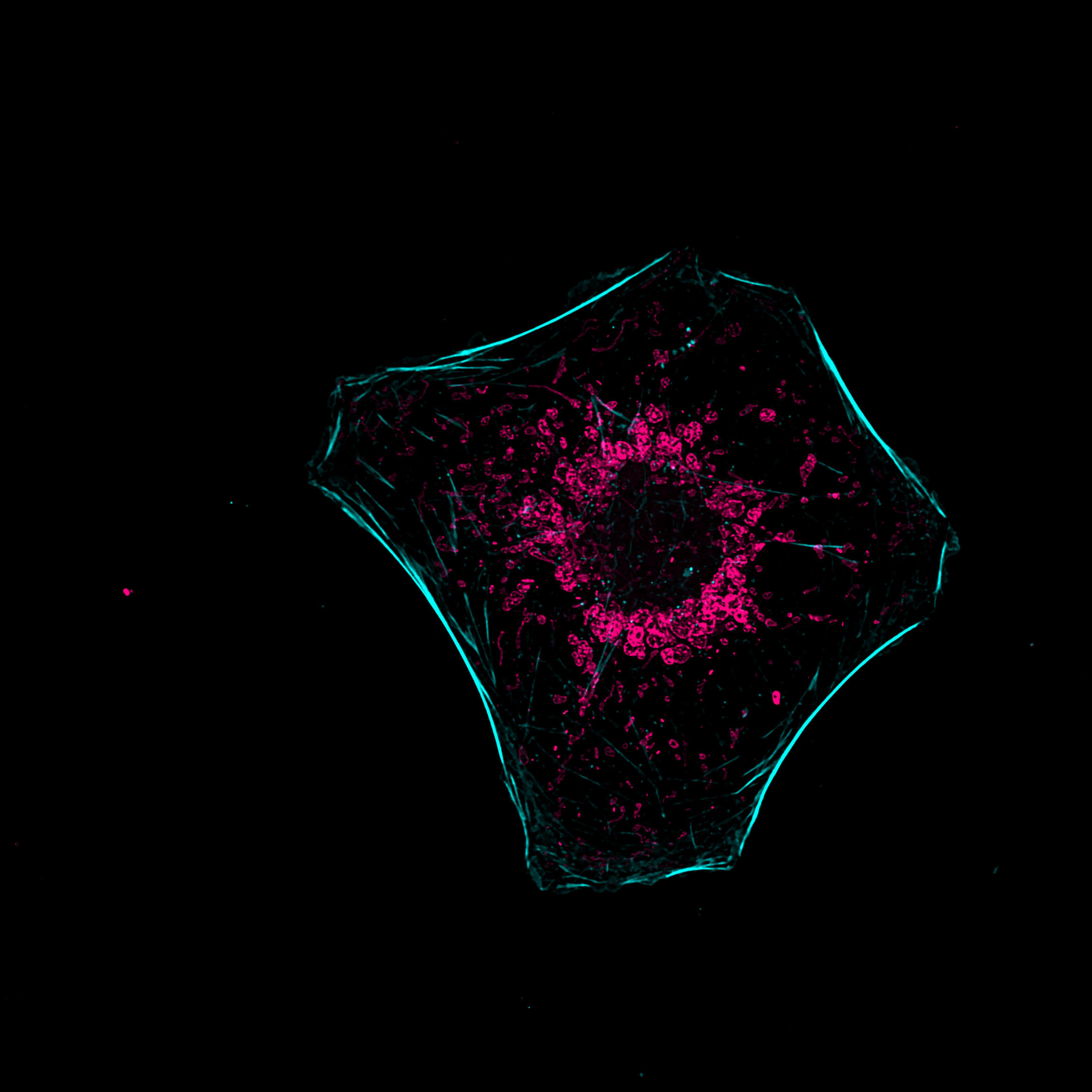

Supplement: Supplementary file 7 — Figure EV1-3 Source Data [file 44319_2024_209_MOESM7_ESM.zip › Figure EV2/EV2F/PFN1 KO TOM20 F-actin.tif]

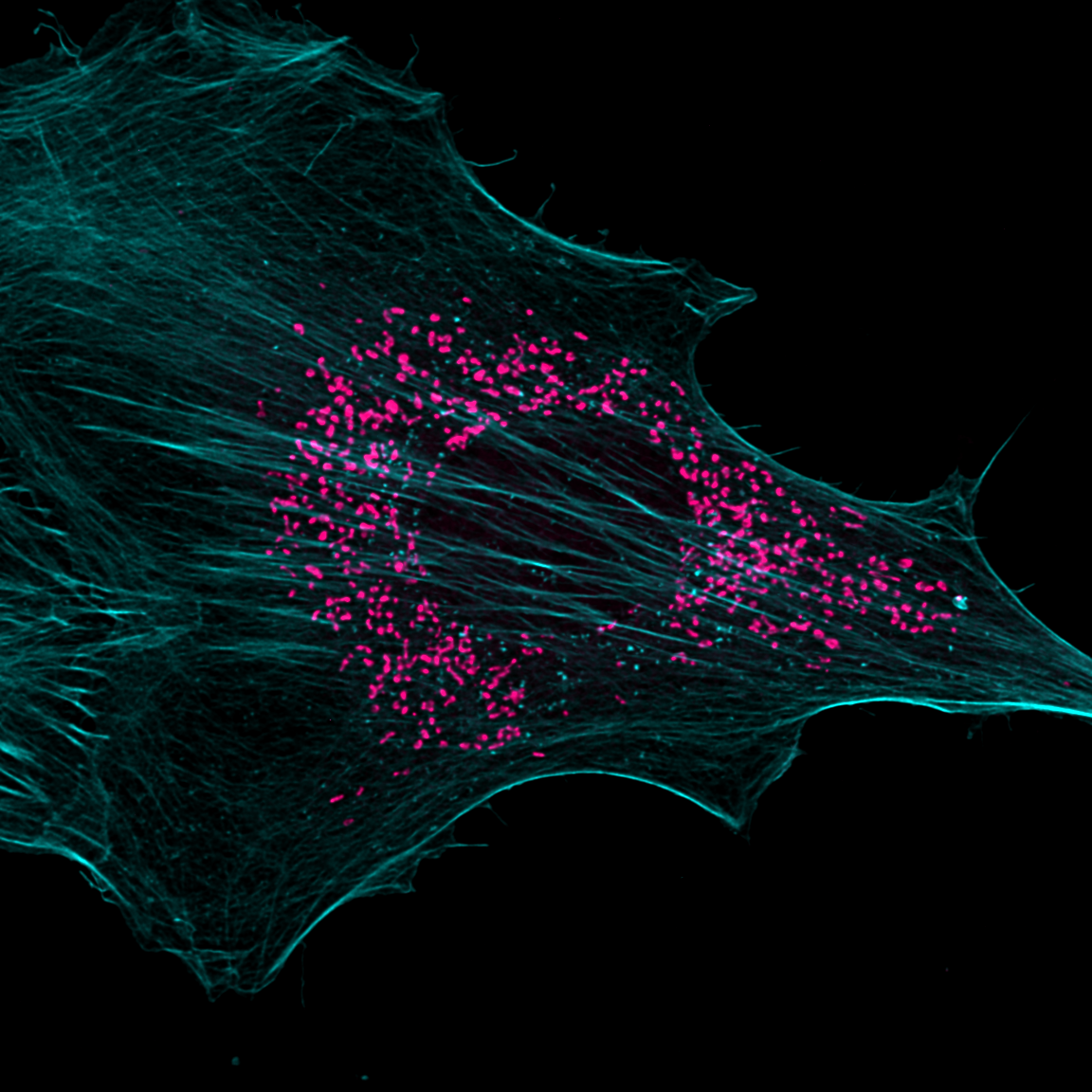

Supplement: Supplementary file 7 — Figure EV1-3 Source Data [file 44319_2024_209_MOESM7_ESM.zip › Figure EV2/EV2F/Control MEF TOM20 F-actin.tif]

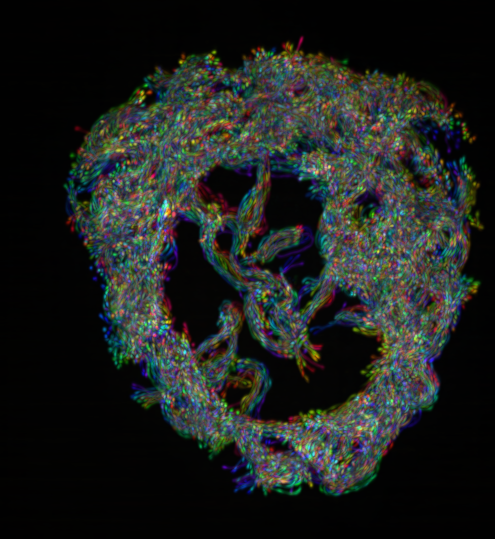

Supplement: Supplementary file 7 — Figure EV1-3 Source Data [file 44319_2024_209_MOESM7_ESM.zip › Figure EV2/EV2A/PFN1 KO time projection.tif]

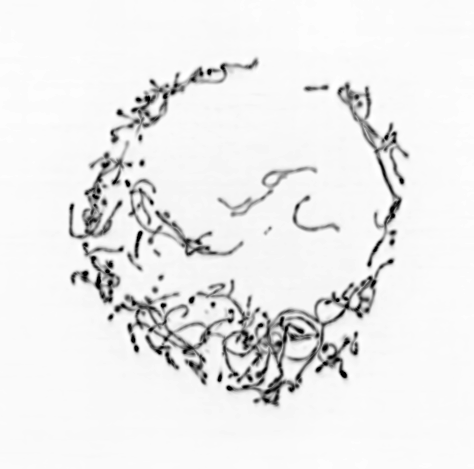

Supplement: Supplementary file 7 — Figure EV1-3 Source Data [file 44319_2024_209_MOESM7_ESM.zip › Figure EV2/EV2A/Control 4xmts-mNeonGreen.tif]

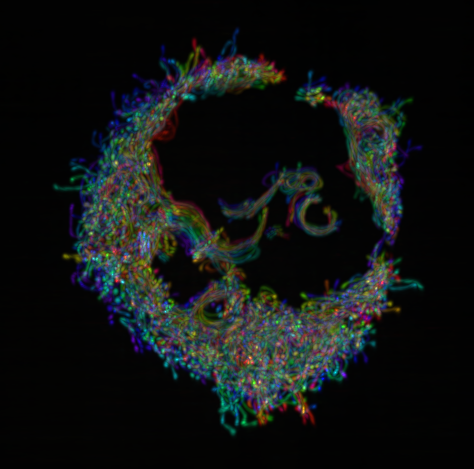

Supplement: Supplementary file 7 — Figure EV1-3 Source Data [file 44319_2024_209_MOESM7_ESM.zip › Figure EV2/EV2A/Control time projection.tif]

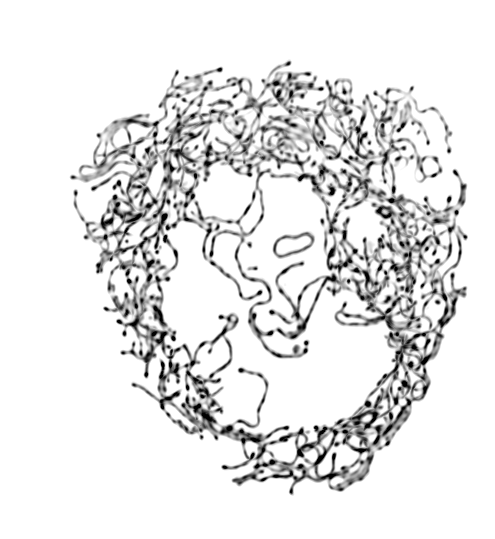

Supplement: Supplementary file 7 — Figure EV1-3 Source Data [file 44319_2024_209_MOESM7_ESM.zip › Figure EV2/EV2A/PFN1 KO 4xmts-mNeonGreen.tif]

**Fig. EV3A**

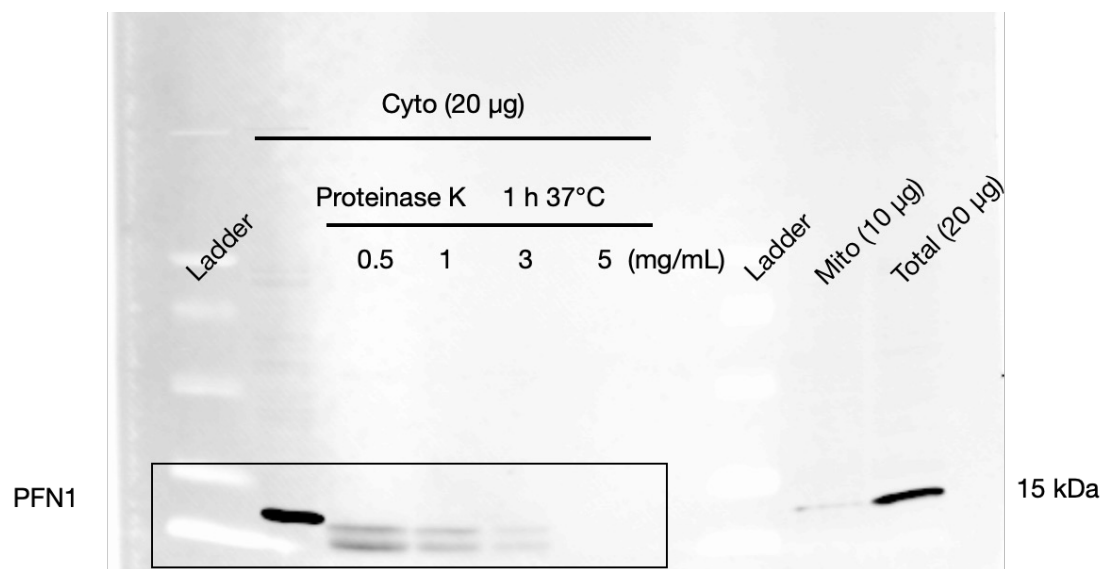

Supplement: Supplementary file 7 — Figure EV1-3 Source Data [file 44319_2024_209_MOESM7_ESM.zip › Figure EV3/EV3A western.pdf]

**Fig. EV3B**

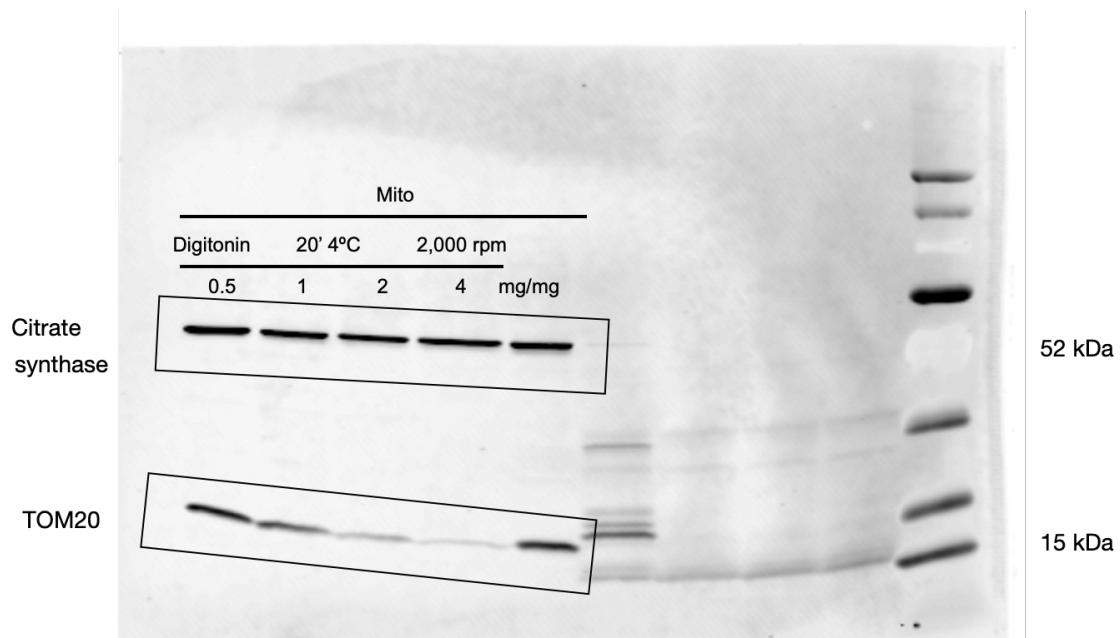

Supplement: Supplementary file 7 — Figure EV1-3 Source Data [file 44319_2024_209_MOESM7_ESM.zip › Figure EV3/EV3B western.pdf]
