# Supplementary material for: The actin binding protein profilin 1 localizes inside mitochondria and is critical for their function
Source: EMBO Rep. 2024 Jul 18;25(8):8. doi: 10.1038/s44319-024-00209-3 (PMC11316047; doi:10.1038/s44319-024-00209-3)
Supplement: Supplementary file 8 — Expanded View Figures [file 44319_2024_209_MOESM8_ESM.pdf]

Expanded View Figures

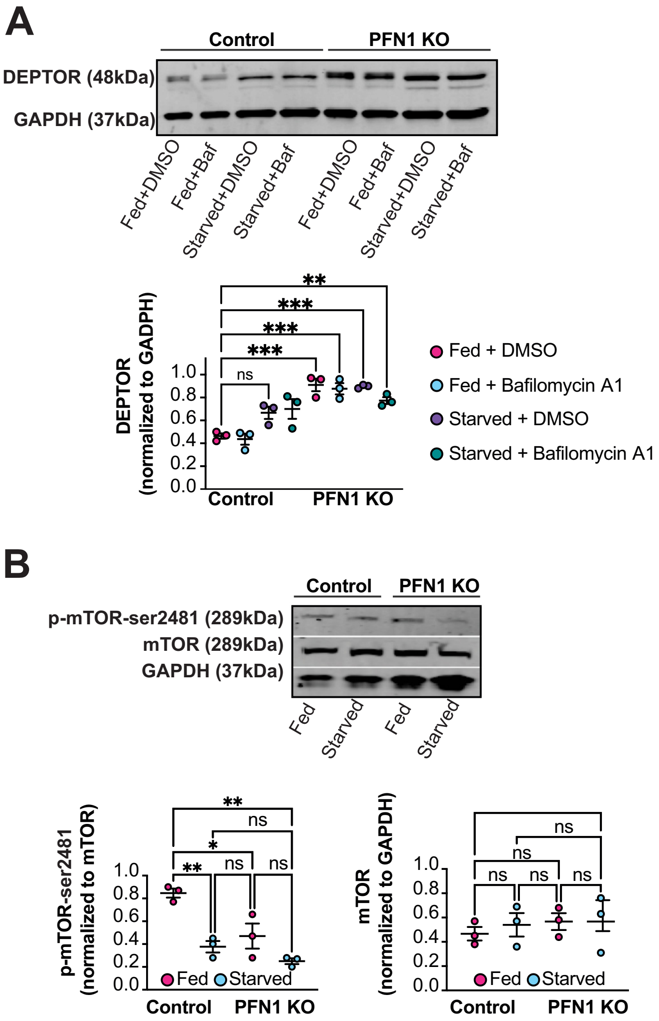

Figure EV1. Loss of PFN1 causes an upregulation of autophagy.

Related to Fig. 1. (**A**) Western blot of DEPTOR and GAPDH in Control and PFN1 KO cells (top). Cells were given normal media (fed) or were nutrient-deprived (starved) for 6 h and were treated with Bafilomycin A (Baf) to inhibit lysosome-mediated degradation or DMSO vehicle control for 4 h. Quantification of DEPTOR normalized against GAPDH (bottom). Data is shown as mean ± SEM and each data point is one biological replicate ($n = 3$). Significance was calculated using ANOVA and Tukey's post hoc test. (**B**) Western blot of mTOR, phospho-S2481-mTOR (p-mTOR-ser2481), and GAPDH in control and PFN1 KO cells (top). Cells were given normal media (fed) or were nutrient-deprived (starved) for 6 h. Quantification of phospho-S2481-mTOR (p-mTOR) (bottom left) and mTOR (bottom right) normalized against GAPDH. Data is shown as mean ± SEM and each data point is one biological replicate ($n = 3$). Significance was calculated using ANOVA and Tukey's post hoc test. Data information: ***$p < 0.001$, **$p < 0.01$, *$p < 0.05$, ns $p > 0.05$. Source data are available online for this figure

© The Author(s)

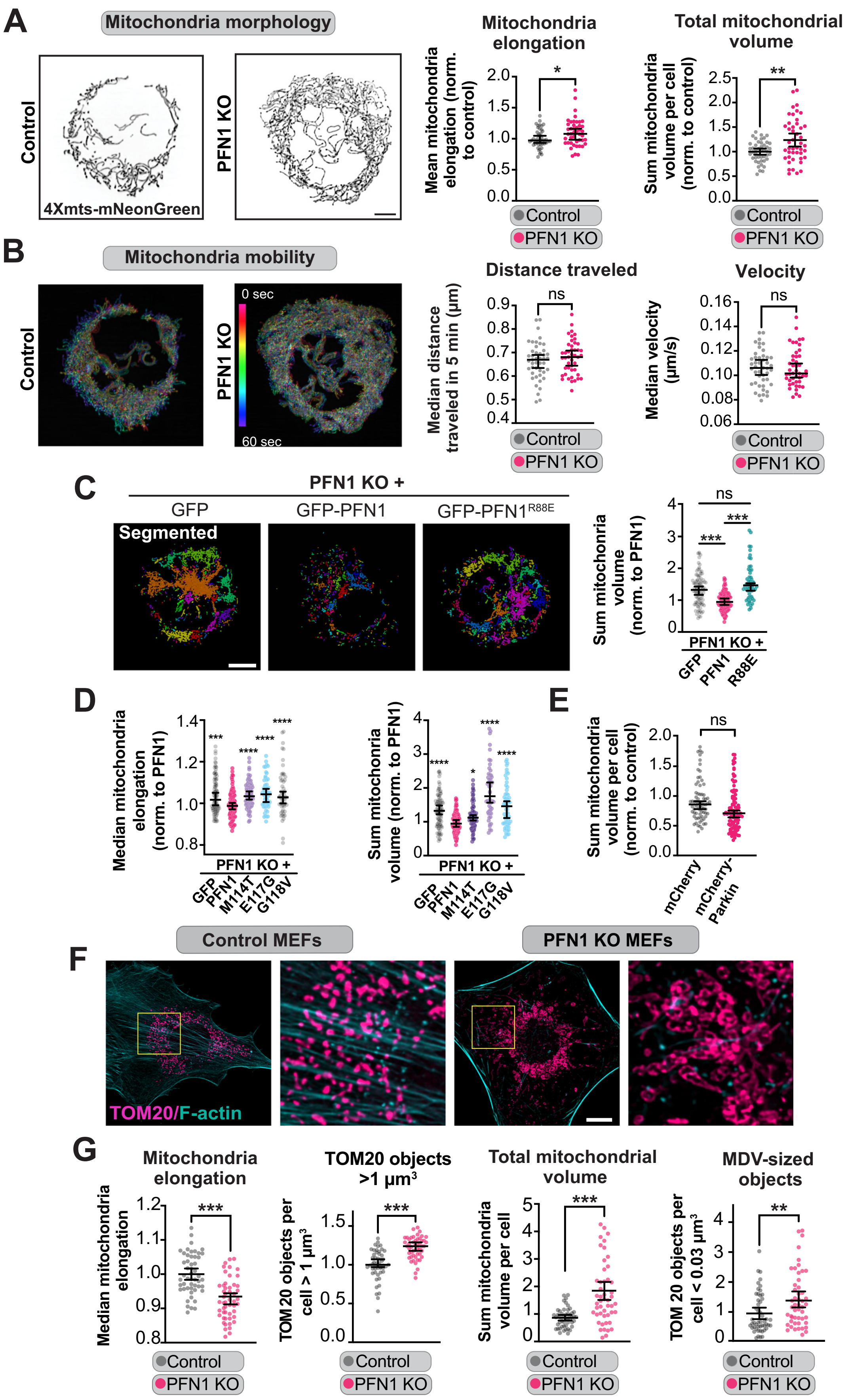
A
Mitochondria morphology
Control
PFN1 KO
4Xmts-mNeonGreen
Mitochondria elongation
Mean mitochondria elongation (norm. to control)
Total mitochondrial volume
Sum mitochondria volume per cell (norm. to control)
Control
PFN1 KO
B
Mitochondria mobility
0 sec
60 sec
Distance traveled
Median distance traveled in 5 min (μm)
Velocity
Median velocity (μm/s)
ns
C
PFN1 KO +
GFP
GFP-PFN1
GFP-PFN1 R88E
Segmented
Sum mitochondria volume (norm. to PFN1)
GFP PFN1 R88E
D
Median mitochondria elongation (norm. to PFN1)
Sum mitochondria volume (norm. to PFN1)
GFP PFN1 M114T E117G G118V
E
Sum mitochondria volume per cell (norm. to control)
mCherry
mCherry-Parkin
F
Control MEFs
PFN1 KO MEFs
TOM20/F-actin
G
Mitochondria elongation
Median mitochondria elongation
TOM20 objects >1 μm³
TOM 20 objects per cell > 1 μm³
Total mitochondrial volume
Sum mitochondria volume per cell
MDV-sized objects
TOM 20 objects per cell < 0.03 μm³

Figure EV2. Loss of PFN1 disrupts mitochondrial morphology.

Related to Fig. 4. (**A**) Representative maximum intensity projections of Control and PFN1 KO cells expressing 4xmts-mNeonGreen (left). Quantification (right) of median mitochondria elongation and sum mitochondria volume in Control and PFN1 KO cells from live cell imaging experiments depicted in (**A**). Each data point represents one cell, in the case of mitochondria elongation, a data point represents the median elongation value of all mitochondria measured from one cell. ($n = 45$ cells for Control and PFN1 KO). Data is shown as median ± 95% CI. Significance was calculated using a Student's t test. and time projections from the entire movie (bottom). (**B**) Time projections from live cell imaging of Control and PFN1 KO cells expressing 4xmts-mNeonGreen, color-coded according to the scale inserted in the right image (left). Quantification of median average velocity and median distance traveled of mitochondria (right). Each data point represents the median of all measurements made in one cell. ($n = 45$ cells for Control and PFN1 KO). Significance was calculated using a Student's t test. Scale bar = 10 µm. (**C**) Representative images of the segmented TOM20 labeled mitochondria in PFN1 KO cells expressing GFP, GFP-PFN1 or the non-actin binding mutant GFP-PFN1^{R88E} (left). Quantification of sum mitochondria volume. Each data point represents the sum mitochondria volume from one cell ($n = 100$ cells for GFP, $n = 97$ for GFP-PFN1, and $n = 56$ for GFP-PFN1^{R88E}). Data is shown as median ± 95% CI. Significance was calculated using a Kruskal–Wallis test followed by Dunn's multiple comparisons test. Scale bar = 10 µm. (**D**) Quantification of sum mitochondria and median mitochondria elongation in PFN1 KO cells expressing either GFP, GFP-PFN1, or the ALS-linked mutations GFP-PFN1^{M114T}, GFP-PFN1^{E117G} and GFP-PFN1^{G118V}. Mitochondria elongation is measured by the length of the longest axis of a 3D object divided by the average of the two smaller axes. Each data point represents one cell, in the case of mitochondria elongation, a data point represents the median elongation value of all mitochondria measured from one cell. ($n = 100$ cells for GFP, $n = 97$ for GFP-PFN1, $n = 77$ for GFP-PFN1^{M114T}, $n = 52$ for GFP-PFN1^{E117G}, $n = 59$ for GFP-PFN1^{G118V}). Data is shown as median ± 95% CI. Significance was calculated using a Kruskal–Wallis test followed by Dunn's multiple comparisons test. (**E**) Quantification of sum mitochondria volume of control cells expressing either mCherry or mCherry-Parkin for 48 h. Each data point represents the sum mitochondria volume from one cell ($n = 75$ for Control and $n = 103$ for PFN1 KO). Data is shown as median ± 95% CI. Significance was calculated using a Mann–Whitney test. (**F**) Representative images of TOM20 labeled mitochondria and F-actin in Control and PFN1 KO cells mouse embryonic fibroblasts (MEFs) (top). Scale bar = 10 µm. (**G**) Quantification of median mitochondria elongation, total mitochondria volume, the number of TOM20 objects >1 µm^3 per cell, and the number of mitochondria-derived vesicle (MDV) sized objects (TOM20 objects <0.03 µm^3) per cell from (**E**). Mitochondria elongation is measured by the length of the longest axis of a 3D object divided by the average of the two smaller axes. Each data point represents one cell, in the case of mitochondria elongation, a data point represents the median elongation value of all mitochondria measured from one cell. ($n = 51$ cells for Control and PFN1 KO). Data is shown as median ± 95% CI. Significance was calculated using a Mann–Whitney test, except for MDV sized objects which used a Student's t-test. Data information: ****$p < 0.0001$, ***$p < 0.001$, **$p < 0.01$, *$p < 0.05$, ns $p > 0.05$. Source data are available online for this figure

 © The Author(s)

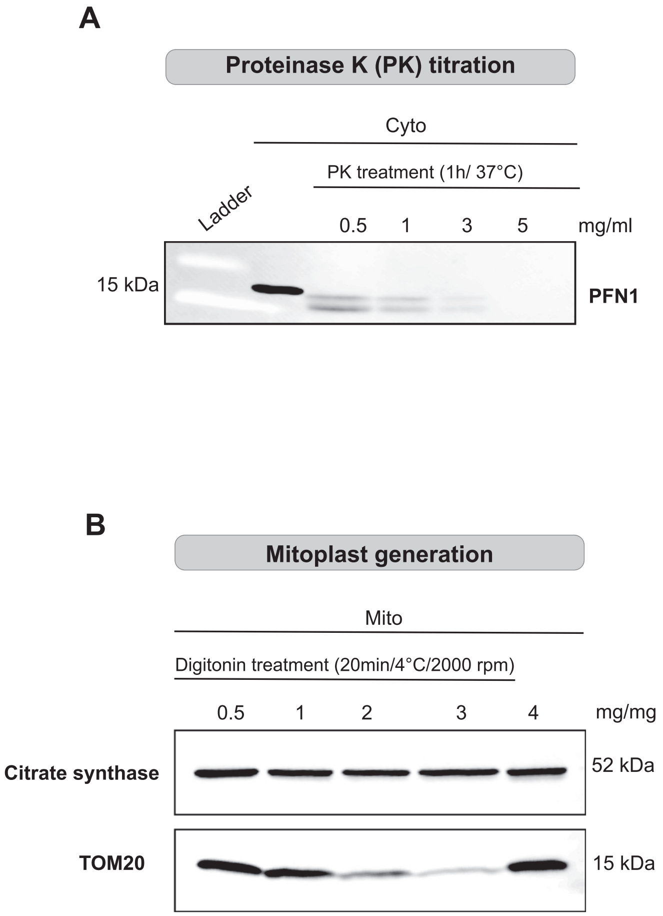

Figure EV3. PFN1 is present inside mitochondria.

Related to Fig. 5. (**A**) Western blot showing titration of Proteinase K applied to the cytoplasmic fraction (Cyto) of CAD cells to identify the optimal concentration at which all cytoplasmic PFN1 is successfully digested. (**B**) Western Blot showing TOM20 (OMM protein) and Citrate synthase (matrix protein) levels from mitochondria treated with increasing amounts of Digitonin to dissolve the OMM to generate mitoplasts. Source data are available online for this figure.

© The Author(s)
